# Prevalence of obesity, hypertension, and diabetes, and cascade of care in sub-Saharan Africa: a cross-sectional, population-based study in rural and urban Malawi

**DOI:** 10.1016/S2213-8587(17)30432-1

**Published:** 2018-03

**Authors:** Alison J Price, Amelia C Crampin, Alemayehu Amberbir, Ndoliwe Kayuni-Chihana, Crispin Musicha, Terence Tafatatha, Keith Branson, Debbie A Lawlor, Elenaus Mwaiyeghele, Lawrence Nkhwazi, Liam Smeeth, Neil Pearce, Elizabeth Munthali, Beatrice M Mwagomba, Charles Mwansambo, Judith R Glynn, Shabbar Jaffar, Moffat Nyirenda

**Affiliations:** a Department of Infectious Disease Epidemiology, London School of Hygiene & Tropical Medicine, London, UK; b Department of Non-Communicable Disease Epidemiology, London School of Hygiene & Tropical Medicine, London, UK; c Malawi Epidemiology and Intervention Research Unit, Lilongwe and Karonga, Malawi; d Medical and Research Department, Dignitas International, Zomba, Malawi; e MRC Integrated Epidemiology Unit and School of Social and Community Epidemiology Medicine, University of Bristol, Bristol, UK; f Ministry of Health, Lilongwe, Malawi; g Global Health Implementation Program, School of Public Health and Family Medicine, College of Medicine, Blantyre, Malawi; h Lighthouse Trust, Kamuzu Central Hospital, Lilongwe, Malawi; i Department of International Public Health, Liverpool School of Tropical Medicine, Liverpool, UK

## Abstract

**Background:**

Sub-Saharan Africa is in rapid demographic transition, and non-communicable diseases are increasingly important causes of morbidity and mortality. We investigated the burden of diabetes, overweight and obesity, hypertension, and multimorbidity, their treatment, and their associations with lifestyle and other factors in Malawi, a very poor country with a predominantly rural—but rapidly growing urban—population, to identify high-risk populations and inform appropriate interventions.

**Methods:**

In this cross-sectional, population-based study, we enrolled all adults (≥18 years) residing in two defined geographical areas within Karonga District and Lilongwe city. All adults self-defining as usually resident in the study areas were eligible, and recruited at household level. Participants were interviewed, had anthropometry and blood pressure measured, and had fasting blood samples collected. The study outcomes were prevalence estimates and risk ratios for diabetes (defined as fasting blood glucose of at least 7·0 mmol/L or self-report of a previous diagnosis of diabetes), hypertension (systolic blood pressure of at least 140 mm Hg, diastolic blood pressure of at least 90 mm Hg, or self-report of current antihypertensive medication), overweight (BMI of 25·0–29·9 kg/m^2^) and obesity (BMI of 30·0 kg/m^2^ or more), and multimorbidity (two or more of the above conditions) by location-specific (urban *vs* rural), age-specific, and sex-specific groups, calculated using negative binomial regression. We used χ^2^ likelihood ratio tests to assess heterogeneity by age, location, and sex.

**Findings:**

Between May 16, 2013, and Feb 8, 2016, we enrolled 15 013 (62%) of 24 367 eligible urban adults in Lilongwe and 13 878 (88%) of 15 806 eligible rural adults in Karonga District. Overweight and obesity, hypertension, and diabetes were highly prevalent, more so in urban residents, the less poor, and better educated than in rural, the poorest, and least educated participants. 18% of urban men (961 of 5211 participants) and 44% (4115 of 9282) of urban women, and 9% (521 of 5834) of rural men and 27% (2038 of 7497) of rural women were overweight or obese; 16% (859 of 5212), 14% (1349 of 9793), 13% (787 of 5847), and 14% (1101 of 8025) had hypertension; and 3% (133 of 3928), 3% (225 of 7867), 2% (84 of 5004), and 2% (124 of 7116) had diabetes, respectively. Of 566 participants with diabetes, 233 (41%) were undiagnosed, and of 4096 participants with hypertension, 2388 (58%) were undiagnosed. Fewer than half the participants on medication for diabetes or hypertension had well controlled diabetes (84 [41%] of 207 participants) or blood pressure (440 [37%] of 1183 participants). Multimorbidity was highest in urban women (n=519, 7%).

**Interpretation:**

Overweight and obesity, hypertension, and diabetes are highly prevalent in urban and rural Malawi, yet many patients are undiagnosed and management is limited. Local-evidence-informed multisectoral, innovative, and targeted interventions are needed urgently to manage the already high burden.

**Funding:**

Wellcome Trust.

## Introduction

Globally, the vast majority of years lived with disability, and deaths due to non-communicable disease (NCD), occur in low-income and middle-income countries.^1^, ^2^ In sub-Saharan Africa, NCDs are projected to account for almost half of all deaths by 2030, as deaths from infectious diseases (excluding HIV) fall, fewer children under 5 years of age die, and adults survive into older age with both chronic, communicable diseases and NCDs.^2^ For a region with constrained health-care resources, this changing disease burden poses a current and future challenge for health policy makers and providers and progress toward development goals. Urbanisation and lifestyle factors (eg, tobacco and alcohol consumption,^3^, ^4^ poor diet,^5^ and physical inactivity^6^) are contributing to this rise. Other factors that are common to low-income and middle-income countries, such as chronic infections including HIV,^7^, ^8^ household air pollution,^9^ and early-life undernutrition,^10^ might also play a part.

**Research in context**

**Evidence before this study**

We searched PubMed for articles published between Sept 1, 1997, and Sept 1, 2016, using the MESH search terms “hypertension”, “glucose metabolism disorders”, “body mass index”, “epidemiological studies”, and “Africa”. We also searched the WHO steps survey database for reports from African countries. Data from many African national and community-level cross-sectional prevalence surveys for overweight and obesity, hypertension, and diabetes have been included in numerous systematic reviews and meta-analyses, but few population-level data are available from Malawi, one of the poorest counties in Africa, which has a predominantly rural population but rapidly growing urban populations.

**Added value of this study**

Our findings show that hypertension, diabetes, and overweight and obesity are all highly prevalent in urban and rural Malawian adults from a young age, despite it being a very low-income country affected by undernutrition and food insecurity. Multimorbidity is highly prevalent in the diabetic population in Malawi, yet diabetes is also evident among the non-obese. Urban-dwelling women are more at risk for multimorbidity than rural women or men in either setting, and the high prevalence of overweight and obesity in those aged 35–54 years is comparable to that observed in women in high-income countries. Even in the poorest populations, the less poor have increased risk of hypertension, diabetes, and overweight or obesity compared with the most poor. Most cases of hypertension and diabetes remain undiagnosed, untreated, or inadequately controlled.

**Implications of all the available evidence**

Hypertension, overweight and obesity, and diabetes are already highly prevalent in Africa. These diseases, which have traditionally been associated with older age, affluence, and urban societies, are increasingly found in young adults, those living in rural locations, and in very low-income countries where access to care is insufficient. Action is needed now to reduce the growing burden of cardiovascular and other non-communicable diseases in Africa. A multisectoral approach is essential, involving government, industry, health-care providers, and the community, to develop evidence-informed context-appropriate strategies for both prevention and treatment of non-communicable diseases.

Reliable, detailed data on NCDs in sub-Saharan Africa remain sparse. We reviewed published studies that reported age-specific or age-standardised prevalence estimates for overweight and obesity, hypertension, and diabetes in sub-Saharan Africa using large, national, or community representative data, many of which have also been included in systematic reviews and meta-analyses.^11^, ^12^, ^13^ A 2009 Malawi NCD Steps survey^14^ showed an already high burden of NCDs despite Malawi being one of the poorest countries in the world.

Our larger study extends this earlier work, providing detailed baseline analyses of blood glucose regulation, by subgroups of age, location (urban *vs* rural), and multimorbidity, and with research infrastructure in place to conduct longitudinal follow-up of participants' access to health-care, health outcomes, and vital statistics in future. We address the essential first step of the WHO Action Plan to prevent and control NCDs: mapping the emerging epidemics and their determinants.^15^ Here, we investigated the prevalence of overweight and obesity, hypertension, diabetes, and multimorbidity, and explored variability by area of residence (urban *vs* rural), age, sex, and other risk factors, to identify high-risk populations and interacting risk factors to help inform the development of new interventions. We also examined participants' self-reported access to a prior diagnosis and treatment for hypertension and diabetes and the adequacy of any existing treatment by using study measures of blood pressure and fasting blood glucose (FBG).

## Methods

### Study design and participants

We conducted population-based studies in northern rural Karonga District and Malawi's central capital city, Lilongwe.^16^ The rural survey was nested in the Karonga Health and Demographic Surveillance Site (HDSS),^17^ an area comprising 135 km^2^ and 39 000 individuals (of whom 15 806 were adults aged ≥18 years) in a predominantly subsistence economy. The urban survey was conducted in Lilongwe, Area-25, a high-density, economically mixed residential area of 23 km^2^, with approximately 66 000 individuals in 2008 (of whom 24 367 were adults),^16^ who engage in private and public sector employment.

In Malawi, 65% of the 17 million population live on less than US$1 per day.^18^ In 2014, average life expectancy at birth was 62·7 years.^18^ At least 80% of the population is rural, but urbanisation is increasing at 4·1% per year due to high rural–urban migration and population growth.^18^ Approximately 10% of adults are HIV positive.^19^ Our two study sites have age structures and sex structures comparable to the national rural and urban populations.^20^ Indices measured in national surveys^19^ show that our districts are similar to the national average in most socioeconomic, lifestyle, and health indicators (the main difference being that Karonga and other northern districts have lower child mortality rates than the rest of Malawi). Only 16% of Malawi's population is urban and approximately 40% of urban residents live in Lilongwe.^20^ Karonga HDSS is typical of Malawi's subsistence farming and fishing communities, and Area-25 is typical of rapidly growing areas in Lilongwe.

All adults (≥18 years) who, prior to data collection (during an annual census in the rural Karonga HDSS or during enumeration in the urban Lilongwe study area), self-reported being usually resident were eligible for study inclusion.

Protocols were approved by the Malawi National Health Sciences Research Committee (protocol #1072) and the London School of Hygiene & Tropical Medicine Ethics Committee (protocol #6303).

### Data collection and definitions

For the NCD survey, participants were visited in their homes. Written informed consent was obtained from all participants for interviewer-led questionnaires, anthropometry and blood pressure measures, and a fasting blood sample (collected by a study nurse at a follow-up home visit). Households were revisited at least three times (including weekends) to recruit eligible individuals missed at an earlier visit. The NCD survey design and methods are described in detail elsewhere.^16^ In brief, we modified the WHO STEPS^14^ instrument and questions from the Hyderabad study^21^ to local needs. Questionnaire data included age, ever (and most recent) measurement of blood pressure and blood glucose, previous clinician diagnoses of diabetes and hypertension, current medication use for diabetes and hypertension, HIV status, household assets, education, employment, physical activity, alcohol consumption, and smoking. Patient-held records were consulted to confirm reported diagnoses and medication for diabetes and hypertension. Questionnaires were translated into Chichewa and Chitumbuka and data were collected by interviewers using electronic tablets.

Weight, height, waist circumference, hip circumference, and mid-upper-arm circumference were measured twice by two study staff independently, following removal of shoes and outer clothing, using calibrated Seca scales, stadiometers, and flexible tape measures. We used the mean of the two measurements in analyses. After 30 min of rest, three seated blood pressure measurements, with 5 min rest in between, were collected on the right arm when possible (otherwise collected on the left arm in those with conditions that precluded use of the right arm), using portable sphygmomanometers (OMRON-Healthcare-Co HEM-7211-E-Model-M6; Kyoto, Japan). We used the mean of the last two blood pressure readings.

A morning venepuncture sample (from 0500 h), after a minimum 8 h fast, was collected at the participant's home by a nurse who also provided HIV screening, using a rapid diagnostic test, in consenting individuals. Non-fasted participants were revisited. FBG samples in sodium fluoride tubes were transported on the same day (99·8%) to the on-site project laboratory for processing (mean time between collection and processing of 2·6 h). We used a hexokinase glucose-6-phosphate dehydrogenase method (Beckman Coulter AU480 Chemistry Analyser, Johannesburg, South Africa), with sufficient sensitivity (range 0·555–44·4 mol/L) to determine glucose concentrations in the study samples. Beckman Coulter controls were tested daily, before sample assays and after recalibration or a change of reagents. The mean intrabatch and interbatch coefficients of variation were less than 3% and 10%, respectively. Our laboratories participated in internal quality control (exchanging samples between sites for repeat testing) and external quality control (Thistle QA, Hohannesburg, South Africa), maintaining coefficients of variation below the external laboratory's acceptable cutoff (<7·4%).^22^

FBG was measured even in those who reported a previous diabetes diagnosis or treatment. We did HbA_1c_ testing in all participants with known diabetes or FBG greater than 5·6 mmol/L, plus a 10% random sample of those with FBG of 5·6 mmol/L or less, using venepuncture samples which were collected at the same visit. In a further 1000 consecutively sampled participants who did not have a previous diagnosis of diabetes, we did HbA_1c_ and oral glucose tolerance tests.

We defined hypertension as systolic blood pressure of at least 140 mm Hg, diastolic blood pressure of at least 90 mm Hg, or self-report of current antihypertensive medication use. Mild hypertension was defined as systolic blood pressure of 140–159 mm Hg or diastolic blood pressure of 90–99 mm Hg; moderate as systolic blood pressure of 160–179 mm Hg or diastolic blood pressure of 100–109 mm Hg; and severe as systolic blood pressure of at least 180 mm Hg or diastolic blood pressure of at least 110 mm Hg. We defined diabetes as FBG of at least 7·0 mmol/L or self-report of a previous diagnosis of diabetes by a health professional, regardless of whether or not the participant was taking antidiabetic medication. Impaired FBG was defined as FBG of at least 6·1 mmol/L, and no more than 6·9 mmol/L in those participants not taking medication. We defined overweight as BMI of 25·0–29·9 kg/m^2^ and obesity as BMI of 30·0 kg/m^2^ or more. Central obesity was defined as waist-to-hip ratio of at least 0·95 for men and at least 0·85 for women.^23^ Multimorbidity was defined as the presence of two or more of hypertension, diabetes, and obesity (not including overweight).^24^

Educational attainment was categorised according to the highest level reached in primary school (standards 1–8), secondary school, or post-secondary education (including vocational training). We collected occupation data in pre-coded categories: government employee, non-government employee, subsistence farmer or fisherman, self-employed, full-time student, at home doing housework, unemployed (but able to work), unemployed (unable to work), and retired. We grouped these categories further into salaried, not working, subsistence, housework, and self-employed groups, and generated proxy wealth scores using locally determined monetary values of household assets, categorised into fifths across the total study population.

We combined self-reported physical exercise duration (minutes) and intensity (pre-coded activities, grouped into high exertion, low exertion, or sedentary) in the previous week (both at work and during leisure time) to generate average metabolic equivalent of task (MET) data per day, and categorised these into whether or not the WHO recommendations of at least 600 Total Physical Activity MET minutes per week were met.^25^ Current smokers were those who reported still smoking or who had stopped within the previous 6 months. Alcohol use was defined as any reported alcohol consumption in the previous year. Average daily sugar consumption (teaspoon equivalents, in drinks) was calculated from the reported number of teaspoons of sugar added to each cup of tea or coffee and usual number of cups of tea or coffee per day, and usual number of pre-sweetened drinks (carbonated and local brands) consumed per day. Sugar consumption in drinks was categorised as fewer than six teaspoons or six or more teaspoons per day, as per WHO guidelines.^26^ We used information on the household size, reported frequency of household purchases of a standard measure of plain salt (equivalent to a 50 g bag of salt which was shown during the interview) to estimate daily average per capita home consumption.

Participants who were identified with elevated blood pressure or FBG (or who tested positive for HIV antibodies for the first time) were referred to the study chronic care clinic or local HIV services, as appropriate, irrespective of whether they were currently on treatment.^27^

### Statistical analysis

We used a conceptual framework in which we categorised risk factors for hypertension or diabetes into distal factors (sex, age, urban or rural residence, education, occupation, and assets) and proximal factors (eg, smoking, physical activity), assuming the former influenced the latter (appendix p 2). This framework determined factors for inclusion in the adjusted models, in which we treated distal factors as potential confounders of the relation between proximal risk factors and risk for outcomes.

We investigated associations between sociodemographic and health-related behavioural risk factors stratified by location (urban *vs* rural) and sex. Age, sex, location, and education were specified a priori as sociodemographic risk factors of interest. We applied age-specific rates of hypertension and diabetes to the WHO standard population,^28^ to generate age-standardised population prevalence estimates for comparison between sites and with external populations. A negative binomial regression model with a log-link function was used to calculate risk ratios for overweight and obesity, diabetes, hypertension, and multimorbidity, adjusting for age and sex. We accounted for potential clustering (because recruitment included all household adults, and family members share factors such as socioeconomic status and diet) by calculating robust SEs. Sex-specific analyses, adjusting for age and location, were followed by multiple regression analyses, adjusting for distal risk factors. If FBG was not available and there was no self-reported diabetes diagnosis, participants were excluded from calculation of diabetes prevalence. For all predefined adjustment variables, missing values represented less than 5% of the data in every variable (pregnant women were excluded from anthropometric analyses). We used χ^2^ likelihood ratio tests to assess heterogeneity by age, location, and sex, and, where observed, we present stratified analyses.

### Role of the funding source

The study funder had no role in the study design; collection, analysis, and interpretation of data; or report writing. The corresponding author had full access to the data and the final responsibility to submit for publication.

## Results

**Figure 1:**
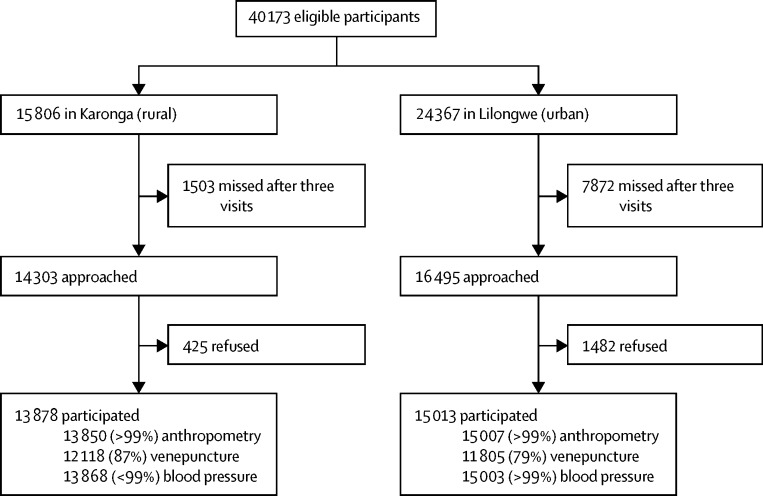
Flow chart of study participation by site

Between May 16, 2013, and Feb 8, 2016, a total of 40 173 individuals were approached (15 806 from Karonga and 24 367 from Lilongwe), of whom 28 891 (72%) enrolled: 13 878 from Karonga and 15 013 from Lilongwe (figure 1), with more women (17 829 [62%]) than men. In the rural area, 10 411 (75%), 2649 (19%), and 871 (6%) participants were recruited on the first, second, and third household visits, respectively; in the urban area, the numbers were 11 505 (77%), 2503 (17%), and 1005 (7%). In the urban area, interviews with 1962 (20%) women and 1532 (29%) men were conducted at weekends. Blood samples from 12 244 (99%) of 12 370 participants were collected before 0800 h in the rural population and from 10 917 (90%) of 12 087 participants before 0900 h in the urban population, where times were recorded.

The median age of the participants was higher in the rural area, partially due to under-recruitment of younger urban men (table 1, appendix p 3). Rural participants had lower levels of education and were poorer than urban residents (table 1). Women had lower levels of education than men. Most rural residents included in the study were subsistence farmers, and a high proportion of urban residents were not employed (table 1).

**Table 1:** Sociodemographic characteristics of participants in rural and urban survey sites

		**Total (N=28 891)**		**Karonga (rural; n=13 878)**		**Lilongwe (urban; n=15 013)**	
		Men (n=11 062)	Women (n=17 829)	Men (n=5849)	Women (n=8029)	Men (n=5213)	Women (n=9800)
Study participant median age, years		32·1 (23·3–44·2)	31·4 (24·1–41·6)	34·3 (24·8–47·4)	34·7 (25·7–48·4)	29·9 (22·4–39·9)	29·7 (23·4–37·7)
Estimated population median age, years*		··	··	34·4	35·0	34·4	29·9
Age group, years							
	18–29	4873 (44%)	8003 (45%)	2249 (38%)	2943 (37%)	2624 (50%)	5016 (51%)
	30–39	2721 (25%)	4742 (27%)	1420 (24%)	1981 (25%)	1301 (25%)	2761 (28%)
	40–49	1519 (14%)	2256 (13%)	904 (15%)	1231 (15%)	615 (12%)	1025 (10%)
	50–59	907 (8%)	1387 (8%)	576 (10%)	835 (10%)	331 (6%)	552 (6%)
	60–69	533 (5%)	814 (5%)	317 (5%)	521 (6%)	216 (4%)	293 (3%)
	≥70	509 (5%)	627 (4%)	383 (7%)	474 (6%)	126 (2%)	153 (2%)
Mean age, years†		36·6 (15·7)	35·3 (14·6)	38·3 (16·5)	38·6 (16·3)	33·5 (13·8)	32·7 (12·5)
Wealth quintiles							
	Poorest	1955 (18%)	3322 (19%)	1425 (24%)	2146 (27%)	530 (10%)	1176 (12%)
	Second	2503 (23%)	3724 (21%)	1918 (33%)	2542 (32%)	585 (11%)	1182 (12%)
	Third	2090 (19%)	3441 (19%)	1163 (20%)	1566 (20%)	927 (18%)	1875 (19%)
	Fourth	2503 (23%)	4158 (23%)	952 (16%)	1257 (16%)	1551 (30%)	2901 (30%)
	Wealthiest	1912 (17%)	3046 (17%)	391 (7%)	518 (6%)	1521 (29%)	2528 (26%)
	Missing data	99 (1%)	138 (1%)	0	0	99 (2%)	138 (1%)
Educational achievement							
	None	173 (2%)	965 (5%)	99 (2%)	496 (6%)	74 (1%)	469 (5%)
	Standard 1–5	886 (8%)	2324 (13%)	635 (11%)	1368 (17%)	251 (5%)	956 (10%)
	Standard 6–8	3195 (29%)	6367 (36%)	2430 (42%)	4088 (51%)	765 (15%)	2279 (23%)
	Secondary	5507 (50%)	6849 (38%)	2513 (43%)	2000 (25%)	2994 (57%)	4849 (49%)
	Post secondary	1301 (12%)	1324 (7%)	172 (3%)	77 (1%)	1129 (22%)	1247 (13%)
Employment							
	Not working	2872 (26%)	2946 (17%)	1037 (18%)	747 (9%)	1835 (35%)	2199 (22%)
	Housework	398 (4%)	5069 (28%)	70 (1%)	767 (10%)	328 (6%)	4302 (44%)
	Subsistence	3397 (31%)	5323 (30%)	3367 (58%)	5278 (66%)	30 (1%)	45 (<1%)
	Self-employed	1912 (17%)	2785 (16%)	851 (15%)	1042 (13%)	1061 (20%)	1743 (18%)
	Salaried	2483 (22%)	1706 (10%)	524 (9%)	195 (2%)	1959 (38%)	1511 (15%)

Data are n (%), median (IQR), or mean (SD).

* Complete data on age were not available for urban residents who did not participate in the study.

† p value for difference in mean age between men and women was 0·0002 in the total population, 0·022 in Karonga (rural), and 0·0002 in Lilongwe (urban).

When investigating modifiable lifestyle risk factors (table 2), we found that rural men were more likely to smoke and drink alcohol than were urban men. Smoking was rare in women, and rural women were less likely to drink alcohol than were urban women. Women were more likely than men to report physical activity levels that met WHO recommendations, as were male rural residents compared with male urban residents. Intake of more than an equivalent of six teaspoons of sugar per day in sweetened drinks was common, particularly among rural men. More than half the individuals in both urban and rural areas lived in households where the per capita consumption of salt used in home cooking or added at the table exceeded 5 g/day.

**Table 2:** Unadjusted prevalence of overweight and obesity, hypertension, diabetes, and lifestyle risk factor categories among study participants

			**Total (N=28 891)**		Karonga (rural; n=13 878)*		Lilongwe (urban; n=15 013)*	
			Men (n=11 062)	Women (n=17 829)	Men (n=5849)	Women (n=8029)	Men (n=5213)	Women (n=9800)
**Overweight and obesity**								
BMI, kg/m^2^								
	<18·0		563 (5%)	584 (3%)	349 (6%)	356 (5%)	214 (4%)	228 (2%)
	18·0–24·9		9000 (81%)	10 042 (56%)	4964 (85%)	5103 (68%)	4036 (77%)	4939 (53%)
	25·0–29·9		1227 (11%)	3908 (22%)	462 (8%)	1467 (20%)	765 (15%)	2441 (26%)
	≥30·0		255 (2%)	2245 (13%)	59 (1%)	571 (8%)	196 (4%)	1674 (18%)
	Unknown or pregnant		17 (<1%)	1050 (6%)	15 (<1%)	532 (7%)	2 (<1%)	518 (5%)
Waist-to-hip ratio†								
	Normal		9019 (82%)	10 597 (59%)	4659 (80%)	3527 (47%)	4360 (84%)	7070 (76%)
	High		2030 (18%)	6191 (35%)	1178 (20%)	3980 (53%)	852 (16%)	2211 (24%)
	Unknown or pregnant		13 (<1%)	1041 (6%)	12 (<1%)	522 (7%)	1 (<1%)	519 (6%)
Mean BMI‡			22·0 (3·2)	24·5 (5·0)	21·6 (2·8)	23·4 (4·3)	22·4 (3·5)	25·4 (5·3)
Mean waist-to-hip ratio‡			0·85 (0·06)	0·83 (0·07)	0·86 (0·05)	0·86 (0·07)	0·84 (0·16)	0·81 (0·07)
**Hypertension**								
Blood pressure: systolic/diastolic, mm Hg								
	Normal or low: <140/90		9413 (85%)	15 368 (86%)	5060 (87%)	6924 (86%)	4353 (84%)	8444 (86%)
	Mild hypertension: 140/90–159/99		970 (9%)	989 (6%)	492 (8%)	452 (6%)	478 (9%)	537 (5%)
	Moderate hypertension: 160/100–179/109		281 (3%)	380 (2%)	135 (2%)	179 (2%)	146 (3%)	201 (2%)
	Severe hypertension: ≥180/110		88 (1%)	199 (1%)	33 (1%)	88 (1%)	55 (1%)	111 (1%)
	On medication		307 (3%)	882 (5%)	127 (2%)	382 (5%)	180 (3%)	500 (5%)
	Unknown		3 (<1%)	11 (<1%)	2 (<1%)	4 (<1%)	1 (<1%)	7 (<1%)
Mean systolic, mm Hg‡			125·9 (15·8)	120·2 (18·2)	124·3 (15·6)	118·9 (19·0)	127·8 (15·7)	121·3 (17·4)
Mean diastolic, mm Hg§			73·6 (10·9)	73·3 (10·8)	73·0 (10·3)	72·6 (10·6)	74·0 (11·5)	73·8 (11·0)
Previous diagnosis of hypertension¶			636 (6%)	1928 (11%)	225 (4%)	656 (8%)	411 (8%)	1272 (13%)
**Diabetes**								
Fasting blood glucose, mmol/L								
	<6·1		8618 (78%)	14 466 (81%)	4868 (83%)	6913 (86%)	3750 (72%)	7553 (77%)
	6·1–6·9		97 (1%)	168 (1%)	52 (1%)	79 (1%)	45 (1%)	89 (1%)
	≥7·0		92 (1%)	141 (1%)	43 (1%)	55 (1%)	49 (1%)	86 (1%)
	Previous diagnosis of diabetes		125 (1%)	208 (1%)	41 (1%)	69 (1%)	84 (2%)	139 (2%)
		On medication	88 (1%)	139 (1%)	33 (1%)	47 (1%)	55 (1%)	92 (1%)
		No medication	37 (<1%)	69 (<1%)	8 (<1%)	22 (<1%)	29 (<1%)	47 (<1%)
	Unknown		2130 (19%)	2846 (16%)	845 (14%)	913 (11%)	1285 (25%)	1933 (20%)
Mean fasting blood glucose, mmol/L**			4·7 (1·2)	4·8 (1·3)	4·7 (1·2)	4·7 (1·9)	4·8 (1·3)	4·8 (1·3)
**Number of conditions (hypertension, obesity, or diabetes)**								
None			7410 (67%)	10 742 (60%)	4262 (85%)	5382 (80%)	3148 (80%)	5360 (71%)
One			1304 (12%)	2695 (15%)	668 (13%)	1058 (16%)	636 (16%)	1637 (23%)
Two			202 (2%)	655 (4%)	64 (1%)	219 (3%)	138 (4%)	436 (6%)
Three			21 (<1%)	110 (1%)	4 (<1%)	27 (<1%)	17 (<1%)	83 (1%)
Unknown			2125 (19%)	3627 (20%)	851 (15%)	1343 (17%)	1274 (33%)	2284 (23%)
**Lifestyle**								
Physical activity (WHO recommendation^25^)								
	Did not meet		676 (6%)	380 (2%)	245 (4%)	187 (2%)	431 (8%)	193 (2%)
	Met recommended		10 386 (94%)	17 449 (98%)	5604 (96%)	7842 (98%)	4782 (92%)	9607 (98%)
	Unknown		0	0	0	0	0	0
Smoking								
	Not current		9801 (89%)	17 791 (>99%)	5060 (87%)	8015 (>99%)	4741 (91%)	9776 (>99%)
	Current		1261 (11%)	38 (<1%)	789 (14%)	14 (<1%)	472 (9%)	24 (<1%)
	Unknown		0	0	0	0	0	0
Alcohol consumption								
	Not in last year		6667 (60%)	16 929 (95%)	3394 (58%)	7739 (96%)	3273 (63%)	9190 (94%)
	In last year		4395 (34%)	900 (5%)	2455 (42%)	290 (4%)	1940 (37%)	610 (6%)
	Unknown		0	0	0	0	0	0
Sugary drinks intake, estimated teaspoons of sugar per day								
	Fewer than six		5152 (47%)	9894 (55%)	2659 (49%)	4544 (64%)	2493 (56%)	5350 (64%)
	Six or more		4719 (43%)	5536 (31%)	2764 (51%)	2531 (36%)	1955 (44%)	3005 (36%)
	Unknown		1191 (11%)	2399 (13%)	426 (7%)	954 (12%)	765 (15%)	1445 (15%)
Average daily per capita plain salt usage in household								
	<2·5 g		1874 (17%)	1855 (10%)	1274 (22%)	1150 (15%)	600 (12%)	705 (7%)
	2·5–5·0 g		3556 (32%)	6620 (37%)	1501 (26%)	2518 (32%)	2055 (40%)	4102 (42%)
	5·1–7·5 g		2768 (25%)	4943 (28%)	1278 (22%)	1914 (24%)	1490 (22%)	3029 (31%)
	>7·5 g		2761 (25%)	4286 (24%)	1707 (30%)	2330 (29%)	1054 (20%)	1956 (20%)
	Unknown		103 (1%)	125 (1%)	89 (2%)	117 (1%)	14 (<1%)	8 (<1%)

Data are n (%) and mean (SD). p values for differences between male and female participants are presented for continuous variables only. Overweight is defined as BMI of 25·0–29·9 kg/m^2^ and obesity as BMI ≥30·0 kg/m^2^. Hypertension is defined as systolic blood pressure ≥140 mm Hg, diastolic blood pressure ≥90 mm Hg, or self-reported current antihypertensive medication use, with in-study blood pressure reading required. Diabetes is defined as fasting blood glucose of ≥7·0 mmol/L or self-report of a previous diagnosis of diabetes by a health professional, regardless of whether or not the participant was taking antidiabetic medication.

* Missing data (unknown) are presented as number and percentage of enrolled participants, and are excluded from the denominator for the calculation of the rural and urban prevalence estimates.

† High waist-to-hip ratio defined as ≥0·95 for male participants and ≥0·85 for female participants.

‡ p value for difference in mean BMI, waist-to-hip ratio, and systolic blood pressure between men and women was <0·0001 in the total population, Karonga (rural), and Lilongwe (urban).

§ p value for difference in mean diastolic blood pressure between men and women was 0·059 in the total population, 0·017 in Karonga (rural), and 0·21 in Lilongwe (urban).

¶ Calculated on full dataset.

** p value for difference in mean fasting blood glucose between men and women was 0·75 in the total population, 0·63 in Karonga (rural), and 0·40 in Lilongwe (urban).

**Figure 2:**
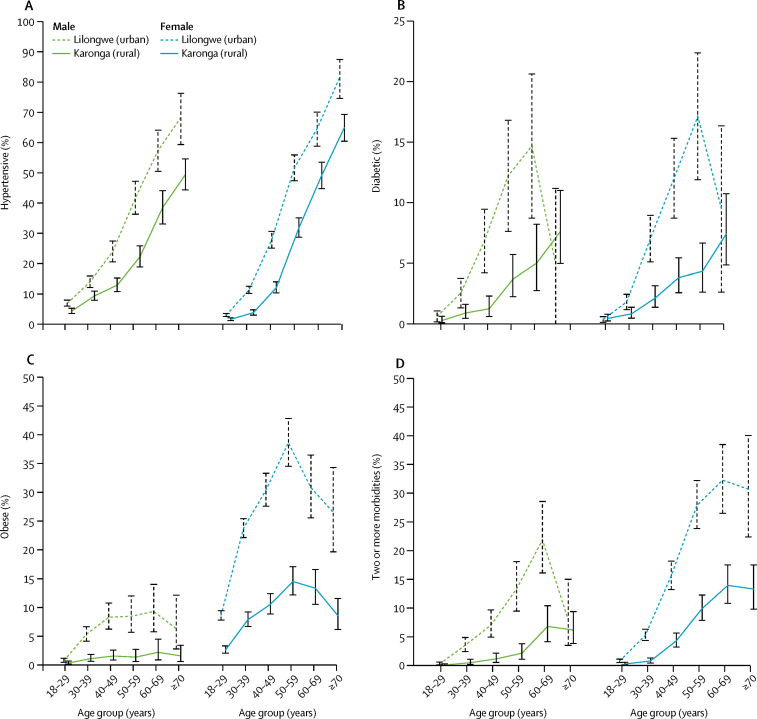
Age-specific and sex-specific prevalence of hypertension, diabetes, obesity, and multimorbidity by site Prevalence point estimates are presented with (vertical) 95% CIs.

Women were more likely to be overweight or obese than were men (table 2). At all ages, the risk of being overweight or obese was higher in urban residents than in rural residents, but most notable in younger age groups (<30 years of age; appendix p 8). When standardised to the WHO world population,^28^ prevalence of overweight and obesity was 40·1% in the urban area and 19·9% in the rural area, representing a national prevalence of 23·1% (weighted for urban *vs* rural population distribution). Overweight and obesity prevalence was greatest in older urban men and rural women (aged 60–69 years) but peaked earlier in urban women (50–59 years), with 70% of participants being overweight or obese (figure 2, appendix p 4). High waist-to-hip ratio was more prevalent in rural residents than in urban residents, despite lower mean BMI (table 2, appendix p 7). When we adjusted for age, the relative risk of overweight and obesity increased with increasing wealth, and, in men, also increased with higher educational attainment, even with distal factor adjustment (table 3). The association of overweight and obesity with increasing wealth was most notable in men aged 30–50 years (appendix p 8). In men, failure to meet the WHO physical activity recommendation and a high intake of sweetened drinks were associated with increased overweight and obesity in the fully adjusted model (table 3). In women, a high intake of sweetened drinks and alcohol consumption were associated with overweight and obesity, but after distal factor adjustment, physical activity was not associated with elevated BMI (table 3).

**Table 3:** Associations of risk factors with overweight and obesity: age-adjusted and distal-factor-adjusted risk ratios

	**Men (n=11 062)**		**Women (n=17 829)**	
	Age adjusted	Distal factor adjusted*	Age adjusted	Distal factor adjusted*
**Site**				
Karonga (rural)	1 (ref)	1 (ref)	1 (ref)	1 (ref)
Lilongwe (urban)	2·41 (2·30–3·04)	1·35 (1·18–1·54)	1·82 (1·74–1·90)	1·45 (1·37–1·55)
**Age group, years**				
18–29	1 (ref)	1 (ref)	1 (ref)	1 (ref)
30–39	2·53 (2·20–2·91)	2·22 (1·90–2·59)	1·69 (1·60–1·78)	1·62 (1·54–1·71)
40–49	3·54 (3·07–4·09)	3·11 (2·65–3·65)	1·77 (1·67–1·88)	1·81 (1·70–1·93)
50–59	3·37 (2·85–3·98)	3·30 (3·77–3·94)	2·02 (1·89–2·15)	2·23 (2·08–2·39)
60–69	3·81 (3·16–4·60)	4·30 (3·56–5·19)	1·90 (1·76–2·06)	2·34 (2·15–2·55)
≥70	3·22 (2·62–3·96)	5·42 (4·37–6·72)	1·30 (1·15–1·46)	1·80 (1·59–2·05)
p value for trend	<0·0001	<0·0001	<0·0001	<0·0001
**Wealth quintiles**				
Poorest	1 (ref)	1 (ref)	1 (ref)	1 (ref)
Second	1·35 (1·08–1·70)	1·26 (1·00–1·58)	1·28 (1·04–1·22)	1·12 (1·04–1·22)
Third	2·12 (1·71–2·62)	1·72 (1·38–2·13)	1·43 (1·33–1·55)	1·28 (1·19–1·40)
Fourth	2·85 (2·33–3·49)	1·98 (1·61–2·43)	1·68 (1·56–1·80)	1·41 (1·31–1·52)
Wealthiest	4·53 (3·72–5·51)	2·70 (2·19–3·34)	2·05 (1·91–2·20)	1·62 (1·50–1·75)
p value for trend	<0·0001	<0·0001	<0·0001	<0·0001
**Education**				
None	0·72 (0·46–1·14)	0·71 (0·46–1·11)	0·94 (0·86–1·03)	0·91 (0·82–1·00)
Standard 1–5	0·79 (0·62–1·00)	0·80 (0·63–1·01)	0·96 (0·90–1·03)	0·95 (0·89–1·02)
Standard 6–8	1 (ref)	1 (ref)	1 (ref)	1 (ref)
Secondary	1·69 (1·49–1·93)	1·22 (1·07–1·40)	1·28 (1·22–1·35)	1·01 (0·96–1·07)
Post secondary	3·56 (3·10–4·08)	1·76 (1·50–2·05)	1·62 (1·52–1·73)	1·08 (1·00–1·17)
p value for trend	<0·0001	<0·0001	<0·0001	0·0542
**Employment**				
Not working	0·70 (0·59–0·82)	0·55 (0·47–0·66)	0·77 (0·72–0·83)	0·70 (0·65–0·75)
Housework	0·59 (0·43–0·81)	0·54 (0·39–0·74)	0·91 (0·86–0·96)	0·86 (0·81–0·90)
Subsistence	0·38 (0·32–0·44)	0·59 (0·50–0·71)	0·51 (0·48–0·55)	0·73 (0·68–0·79)
Self-employed	1 (ref)	1 (ref)	1 (ref)	1 (ref)
Salaried	1·25 (1·11–1·41)	0·96 (0·85–1·08)	1·05 (0·98–1·11)	0·87 (0·81–0·93)
**Physical activity (WHO recommendation**^25^)				
Did not meet	1·96 (1·73–2·23)	1·30 (1·15–1·49)	1·21 (1·08–1·37)	1·07 (0·95–1·20)
Met recommendation	1 (ref)	1 (ref)	1 (ref)	1 (ref)
**Smoking**				
Not current	1 (ref)	1 (ref)	1 (ref)	1 (ref)
Current	0·32 (0·25–0·40)	0·44 (0·35–0·56)	0·52 (0·26–1·04)	0·49 (0·26–0·94)
**Alcohol consumption**				
Not in last year	1 (ref)	1 (ref)	1 (ref)	1 (ref)
In last year	0·97 (0·88–1·07)	1·06 (0·96–1·17)	1·39 (1·29–1·50)	1·26 (1·18–1·37)
**Sugary drinks intake, teaspoons of sugar per day**				
Fewer than six	1 (ref)	1 (ref)	1 (ref)	1 (ref)
Six or more	1·33 (0·20–1·47)	1·20 (1·08–1·32)	1·19 (1·14–1·24)	1·12 (1·07–1·17)

Data are risk ratios (95% CI). Overweight and obesity defined as body-mass index >25 kg/m^2^. Analyses are stratified by sex because we found significant heterogeneity in the association of sex and overweight and obesity by site (p=0·0035) and age (p<0·0001). Further stratified analyses (age-sex) and (site-sex) are presented in the appendix.

* Adjusted for age, site, wealth, education, and occupation (see appendix p 2).

The crude prevalence of hypertension was 14·7% in the urban area and 13·6% in the rural area (table 2). The prevalence, and urban–rural difference, increased markedly when standardised to the WHO world population^25^ (22·5% in the urban area and 14·5% in the rural area), representing a weighted age-standardised national prevalence of 15·8%. The prevalence increased steeply from a young age in both sexes, but the increase started about 10 years earlier in urban residents (figure 2, appendix p 5). A greater proportion of the burden of hypertension in urban residents was in people younger than 50 years of age (1262 [57%] of 2208 urban *vs* 621 [33%] of 1888 rural). Many people with hypertension (900 [41%] of 2208 urban and 1188 [63%] of 1888 rural) were not overweight or obese. Associations between increasing wealth and education were largely attenuated after adjustment for BMI in both men and women (table 4). In older people (ie, >50 years), increasing educational attainment was associated with increased hypertension risk in men but not in women (appendix p 9). The association of hypertension with increasing wealth was seen in middle-aged residents (30–50 years) but not in younger (<50 years) or older residents (>50 years) in either location.

**Table 4:** Associations of risk factors with hypertension: age-adjusted and distal-factor-adjusted risk ratios

	**Male (n=11 062)**			**Female (n=17 829)**		
	Age adjusted	Distal factor adjusted*	Additional BMI adjustment†	Age adjusted	Distal factor adjusted*	Additional BMI adjustment†
**Site**						
Karonga (rural)	1 (ref)	1 (ref)	1 (ref)	1 (ref)	1 (ref)	1 (ref)
Lilongwe (urban)	1·61 (1·48–1·76)	1·38 (1·22–1·55)	1·32 (1·17–1·48)	1·82 (1·70–1·96)	1·37 (1·24–1·51)	1·28 (1·15–1·55)
**Age group, years**						
18–29	1 (ref)	1 (ref)	1 (ref)	1 (ref)	1 (ref)	1 (ref)
30–39	2·01 (1·73–2·35)	2·03 (1·72–2·39)	1·80 (1·53–2·12)	3·27 (2·76–3·86)	3·30 (2·82–3·94)	2·92 (2·45–3·47)
40–49	2·03 (2·58–3·54)	3·06 (2·58–3·62)	2·58 (2·17–3·06)	7·66 (6·52–8·99)	8·29 (7·09–9·85)	6·73 (5·66–8·00)
50–59	5·10 (4·38–5·94)	5·40 (4·60–6·34)	4·57 (3·88–5·38)	15·81 (13·6–18·4)	18·45 (15·7–21·5)	14·14 (11·95–16·27)
60–69	8·03 (6·93–9·29)	8·62 (7·42–10·01)	7·12 (6·10–8·31)	21·76 (18·7–25·3)	26·79 (22·3–31·0)	20·77 (17·54–24·59)
≥70	9·41 (8·20–10·8)	12·0 (10·31–14·03)	10·01 (8·59–11·79)	27·49 (23·7–31·8)	35·10 (29·4–41·4)	29·07 (24·46–34·55)
p value for trend	<0·0001	<0·0001	<0·0001	<0·0001	<0·0001	<0·0001
**Wealth quintiles**						
Poorest	1 (ref)	1 (ref)	1 (ref)	1 (ref)	1 (ref)	1 (ref)
Second	1·09 (0·94–1·29)	1·07 (0·91–1·25)	1·06 (0·90–1·24)	0·92 (0·90–1·13)	1·03 (0·92–1·15)	0·99 (0·88–1·12)
Third	1·34 (1·14–1·57)	1·20 (1·03–1·41)	1·15 (0·98–1·35)	1·25 (1·12–1·40)	1·13 (1·00–1·26)	1·04 (0·92–1·16)
Fourth	1·45 (1·25–1·69)	1·18 (1·01–1·38)	1·09 (0·93–1·28)	1·54 (1·39–1·71)	1·27 (1·13–1·42)	1·14 (1·02–1·28)
Wealthiest	1·84 (1·58–2·15)	1·32 (1·11–1·56)	1·10 (0·93–1·31)	1·79 (1·60–2·00)	1·34 (1·18–1·52)	1·13 (0·99–1·28)
p value for trend	<0·0001	0·0076	0·396	<0·0001	0·0001	0·061
**Education**						
None	0·80 (0·61–1·06)	0·78 (0·60–1·03)	0·78 (0·59–1·04)	1·08 (0·97–1·21)	1·07 (0·96–1·20)	1·12 (1·00–1·26)
Standard 1–5	0·87 (0·74–1·01)	0·87 (0·74–1·02)	0·88 (0·75–1·03)	1·07 (0·97–1·18)	1·09 (1·00–1·20)	1·12 (1·01–1·23)
Standard 5–8	1 (ref)	1 (ref)	1 (ref)	1 (ref)	1 (ref)	1 (ref)
Secondary	1·19 (1·07–1·33)	1·05 (0·94–1·18)	1·00 (0·89–1·12)	1·44 (1·30–1·59)	1·14 (1·02–1·26)	1·11 (0·99–1·24)
Post secondary	1·87 (1·64–2·13)	1·41 (1·22–1·65)	1·23 (1·06–1·44)	1·79 (1·54–2·08)	1·19 (0·99–1·42)	1·13 (0·94–1·36)
p value for trend	<0·0001	0·0001	0·0112	<0·0001	0·647	0·733
**Employment**						
Not working	0·99 (0·85–1·15)	0·90 (0·78–1·05)	0·96 (0·83–1·12)	0·98 (0·86–1·12)	0·95 (0·83–1·08)	0·99 (0·87–1·13)
Housework	0·98 (0·76–1·26)	0·91 (0·71–1·78)	0·99 (0·77–1·27)	1·06 (0·94–1·18)	1·00 (0·89–1·12)	1·04 (0·92–1·16)
Subsistence	0·71 (0·62–0·81)	0·92 (0·79–1·08)	0·99 (0·85–1·16)	0·59 (0·53–0·66)	0·77 (0·68–0·88)	0·85 (0·75–0·97)
Self-employed	1 (ref)	1 (ref)	1 (ref)	1 (ref)	1 (ref)	1 (ref)
Salaried	1·12 (0·98–1·29)	0·97 (0·84–1·11)	0·99 (0·86–1·14)	1·25 (1·08–1·45)	1·04 (0·88–1·22)	1·08 (0·91–1·27)
**Physical activity (WHO recommendation**^25^**)**						
Did not meet	1·35 (1·20–1·51)	1·15 (1·02–1·30)	··	1·33 (1·20–1·47)	1·16 (1·05–1·29)	··
Met recommendation	1 (ref)	1 (ref)	··	1 (ref)	1 (ref)	··
**Smoking**						
Not current	1 (ref)	1 (ref)	··	1 (ref)	1 (ref)	··
Current	0·74 (0·64–0·87)	0·86 (0·73–1·00)	··	0·78 (0·50–1·20)	0·76 (0·49–1·18)	··
**Alcohol consumption**						
Not in last year	1 (ref)	1 (ref)	··	1 (ref)	1 (ref)	··
In last year	1·03 (0·94–1·13)	1·11 (1·01–1·21)	··	1·13 (0·97–1·33)	1·11 (0·95–1·30)	··
**Sugary drinks intake, teaspoons of sugar per day**‡						
Fewer than six	1 (ref)	1 (ref)	··	1 (ref)	1 (ref)	··
Six or more	0·87 (0·79–0·96)	0·84 (0·76–0·93)	··	0·85 (0·78–0·93)	0·79 (0·72–0·87)	··
**BMI, kg/m^2^**						
<18·0	0·75 (0·60–0·94)	0·76 (0·61–0·96)	··	0·87 (0·73–1·05)	0·90 (0·75–1·08)	··
18·0–24·9	1 (ref)	1 (ref)	··	1 (ref)	1 (ref)	··
25·0–29·9	2·13 (1·92–2·36)	1·89 (1·70–2·10)	··	1·63 (1·49–1·78)	1·49 (1·37–1·63)	··
≥30·0	3·44 (3·00–3·95)	2·80 (2·40–3·25)	··	2·41 (2·22–2·63)	2·02 (1·85–2·22)	··
**Waist-to-hip ratio**						
Normal	1 (ref)	1 (ref)	··	1 (ref)	1 (ref)	··
High	1·69 (1·53–1·86)	1·59 (1·44–1·75)	··	1·16 (1·07–1·24)	1·31 (1·23–1·42)	··

Data are risk ratios (95% CI). Hypertension is defined as systolic ≥140 mm Hg, diastolic ≥90 mm Hg, or on antihypertensive medication. We found no evidence of interaction between sex and age or site and risk for hypertension. We found evidence for significant interaction between age and sex (p<0·0001) and age and site (p=0·0003) in their associations with hypertension. Further stratified analyses (age-sex) and (age-site) are presented in the appendix.

* Adjusted for distal factors: age, site, wealth, education, and occupation (see appendix p 2).

† Additional BMI adjustment is added to adjustment for distal factors. We did not do additional BMI adjustment for lifestyle factors prone to change with changes in health and associated with BMI.

‡ After adjusting for distal factors and excluding those with a previous diagnosis of diabetes, the odds ratio for association of at least six teaspoons of sugar daily and newly diagnosed diabetes was 0·78 (0·49–1·23) in men and 1·11 (0·77–1·61) in women.

Of participants older than 40 years of age (and thus considered eligible for annual hypertension screening in other settings, such as the USA^29^) and with a high BMI (ie, overweight or obese), rural men were least likely to report ever having had their blood pressure measured (378 [17%] of 2180 rural men aged over 40 years, of whom 132 [25%] of 536 who were obese; appendix p 10). Only 2277 (56%) of 4096 participants with hypertension had been previously screened; 1708 (42%) were already diagnosed and 1189 (29%) were taking regular antihypertensive medication. Of those on medication, fewer than half had blood pressure below 140/90 mm Hg (440 [37%] of 1183 participants). Urban women were most likely to have accessed screening for hypertension, have a correct diagnosis, take hypertensive medication, and to have adequately controlled blood pressure (appendix p 10). Throughout the cascade of care, rural men were least likely to have been screened, diagnosed, and be on medication (appendix p 10).

The crude prevalence of diabetes was 2·4% overall (3·0% in the urban area and 1·7% in the rural area; table 2); after age-standardisation to WHO population criteria^28^ we found a prevalence of 5·4% in urban areas and 2·1% in rural areas, representing a national prevalence of 2·6% (weighted for urban *vs* rural population distribution). 170 (71%) of 240 participants with raised FBG but no previous diagnosis and 160 (84%) of 191 participants reporting a prior diagnosis had an elevated HbA_1c_. In the sub-sample of 1000 participants, inclusion of those with isolated post-prandial hyperglycaemia would only have increased prevalence of newly diagnosed diabetes by less than 20%. Diabetes prevalence increased with age in both locations (figure 2). Unlike hypertension, the age-specific prevalence of diabetes (and impaired FBG) diverged between rural and urban populations, in both sexes (figure 2, appendix p 6). 80 (38%) of 208 rural and 184 (51%) of 358 urban individuals with diabetes were younger than 50 years of age. A large proportion of people with diabetes (95 [47%] of 204 rural and 91 [26%] of 352 urban) were not overweight or obese, in those with height and weight data. Increased wealth and better education were associated with increased diabetes risk in both men and women; this association was attenuated only slightly by adjusting for BMI (table 5). Smoking and alcohol were not associated with risk for diabetes, but risk increased in those with low physical activity levels (table 5). Diabetes appeared to be associated with a low intake of sweetened drinks, but this relationship disappeared after excluding those who had previously been given a diagnosis of diabetes (table 5), possibly due to patients following the dietary advice given at diagnosis, as recommended in Standard Treatment Clinical Guidelines.^30^

**Table 5:** Associations of risk factors with diabetes: sex-specific crude and adjusted risk ratios

	**Men (n=11 062)**			**Women (n=17 829)**		
	Age adjusted	Distal factor adjusted*	Additional BMI adjustment†	Age adjusted	Distal factor adjusted*	Additional BMI adjustment†
**Site**						
Karonga (rural)	1 (ref)	1 (ref)	1 (ref)	1 (ref)	1 (ref)	1 (ref)
Lilongwe (urban)	2·76 (2·12–3·59)	2·07 (1·42–3·05)	1·87 (1·28–2·74)	2·57 (2·06–3·20)	1·45 (1·04–2·02)	1·24 (0·89–1·72)
**Age group, years**						
18–29	1 (ref)	1 (ref)	1 (ref)	1 (ref)	1 (ref)	1 (ref)
30–39	3·66 (2·05–6·50)	3·86 (2·08–7·16)	3·22 (1·73–5·99)	3·48 (2·19–5·54)	3·52 (2·17–5·70)	2·45 (1·48–4·04)
40–49	7·57 (4·33–13·23)	7·58 (4·17–13·77)	5·87 (3·21–10·74)	10·73 (6·92–16·60)	12·26 (7·61–19·73)	8·01 (4·90–13·10)
50–59	14·83 (8·61–25·54)	17·55 (9·76–31·55)	13·55 (7·51–24·48)	17·56 (11·37–27·14)	21·51 (14·04–36·08)	13·00 (7·90–21·39)
60–69	19·90 (11·40–34·74)	22·17 (12·58–39·08)	16·29 (9·16–28·99)	22·72 (14·47–35·67)	34·00 (21·22–54·50)	19·19 (11·71–31·45)
≥70	15·75 (8·76–28·31)	28·75 (15·45–53·47)	20·98 (11·20–39·27)	20·07 (12·23–32·95)	37·66 (21·67–65·44)	21·85 (12·39–38·51)
p value for trend	<0·0001	<0·0001	<0·0001	<0·0001	<0·0001	<0·0001
**Wealth quintiles**						
Poorest	1 (ref)	1 (ref)	1 (ref)	1 (ref)	1 (ref)	1 (ref)
Second	1·20 (0·65–2·23)	1·10 (0·59–2·03)	1·19 (0·59–2·00)	1·49 (0·94–2·37)	1·43 (0·90–2·28)	1·48 (0·91–2·38)
Third	1·57 (0·86–2·89)	1·25 (0·69–2·28)	1·19 (0·66–2·17)	1·80 (1·14–2·86)	1·48 (0·93–2·35)	1·38 (0·86–2·22)
Fourth	2·73 (1·56–4·79)	1·80 (1·03–3·16)	1·62 (0·92–2·85)	3·17 (2·09–4·80)	2·18 (1·42–3·36)	1·95 (1·24–3·04)
Wealthiest	5·21 (3·03–8·96)	2·73 (1·57–4·76)	2·20 (1·24–3·89)	5·28 (3·52–7·01)	3·07 (1·97–4·77)	2·37 (1·50–3·75)
p value for trend	<0·0001	<0·0001	0·0012	<0·0001	<0·0001	0·0002
**Education**						
None	0·20 (0·028–1·39)	0·20 (0·028–1·43)	0·21 (0·029–1·48)	0·54 (0·34–0·86)	0·61 (0·39–0·97)	0·74 (0·47–1·15)
Standard 1–5	0·73 (0·40–1·33)	0·76 (0·42–1·40)	0·77 (0·42–1·41)	0·67 (0·48–0·92)	0·74 (0·54–1·07)	0·81 (0·59–1·12)
Standard 5–8	1 (ref)	1 (ref)	1 (ref)	1 (ref)	1 (ref)	1 (ref)
Secondary	1·80 (1·27–2·55)	1·40 (0·97–2·04)	1·27 (0·88–1·85)	1·75 (1·34–2·29)	1·12 (0·84–1·50)	1·05 (0·78–1·42)
Post secondary	4·15 (2·87–6·00)	2·16 (1·40–3·34)	1·84 (1·17–2·89)	3·11 (2·25–4·30)	1·44 (0·97–2·15)	1·24 (0·83–1·87)
p value for trend	<0·0001	<0·0001	0·0006	<0·0001	0·0015	0·0552
**Employment**						
Not working	1·12 (0·74–1·69)	0·84 (0·55–1·29	0·92 (0·23–1·39)	1·02 (0·71–1·46)	0·84 (0·59–1·20)	0·87 (0·60–1·25)
Housework	0·52 (0·19–1·45)	0·49 (0·18–1·37)	0·52 (0·19–1·44)	0·75 (0·56–1·01)	0·70 (0·51–0·95)	0·73 (0·54–1·00)
Subsistence	0·56 (0·38–0·83)	1·29 (0·80–2·08)	1·42 (0·88–2·30)	0·36 (0·26–0·50)	0·59 (0·40–0·87)	0·70 (0·47–1·04)
Self-employed	1 (ref)	1 (ref)	1 (ref)	1 (ref)	1 (ref)	1 (ref)
Salaried	1·21 (0·82–1·78)	0·86 (0·58–1·27)	0·91 (0·61–1·35)	1·28 (0·90–1·83)	0·77 (0·52–1·13)	0·82 (0·55–1·22)
**Physical activity (WHO recommendation**^25^**)**						
Did not meet	2·44 (1·77–3·37)	1·53 (1·11–2·13)	··	1·99 (1·32–3·02)	1·55 (1·02–2·36)	··
Met recommendation	1 (ref)	1 (ref)	··	1 (ref)	1 (ref)	··
**Smoking**						
Not current	1 (ref)	1 (ref)	··	1 (ref)	1 (ref)	1 (ref)
Current	0·60 (0·37–0·96)	0·91 (0·56–1·29)	··	0·76 (0·12–5·06)	0·99 (0·19–5·22)	1·38 (0·30–6·29)
**Alcohol consumption**						
Not in last year	1 (ref)	1 (ref)	··	1 (ref)	1 (ref)	1 (ref)
In last year	0·72 (0·54–0·96)	0·87 (0·66–1·18)	··	1·20 (0·75–1·90)	1·22 (0·78–1·91)	1·11 (0·71–1·72)
**Sugary drinks intake, teaspoons of sugar per day**						
Fewer than six	1 (ref)	1 (ref)	··	1 (ref)	1 (ref)	··
Six or more	0·42 (0·30–0·59)	0·37 (0·27–0·52)‡	··	0·89 (0·69–1·14)	0·73 (0·57–0·95)‡	··
**BMI, kg/m^2^**						
<18·0	0·52 (0·21–1·27)	0·57 (0·23–1·39)	··	0·76 (0·27–2·11)	0·81 (0·29–2·25)	··
18·0–24·9	1 (ref)	1 (ref)	··	1 (ref)	1 (ref)	··
25·0–29·9	3·11 (2·35–4·12)	2·15 (1·57–2·93)	··	3·04 (2·25–4·09)	2·50 (1·86–3·37)	··
≥30·0	5·34 (3·59–7·96)	2·76 (1·72–4·42)	··	6·47 (4·89–8·56)	4·43 (3·29–5·97)	··
**Waist-to-hip ratio**						
Normal	1 (ref)	1 (ref)	··	1 (ref)	1 (ref)	··
High	3·86 (2·85–5·24)	3·11 (2·27–4·27)	··	2·11 (1·64–2·70)	2·64 (2·06–3·37)	··

Data are risk ratios (95% CI). We defined diabetes as fasting blood glucose >7·0 mmol/L or previous diagnosis. We found no evidence of interaction between sex and age or site and risk for diabetes. We found evidence for significant interaction between age-site (p=0·043). Additional stratifications are not presented because numbers are small.

* Adjusted for age, site, wealth, education, and occupation (see appendix p 2).

† Additional BMI adjustment is added to adjustment for distal factors. We did not do additional BMI adjustment for lifestyle factors prone to change with changes in health and associated with BMI.

‡ After excluding those with a previous diagnosis, these values became 0·78 (0·49–1·23) for men and 1·11 (0·77–1·61) for women.

Few participants reported ever having had their blood glucose measured (appendix p 11). Of the 566 participants we found had diabetes, just over half (304 [54%]) reported having had previous screening, and 333 (59%) had been previously diagnosed. 227 (68%) of those with a previous diagnosis were taking regular medication; of the 207 who consented to have FBG measured, fewer than half (84 [41%]) had adequate blood glucose control. Of 233 participants diagnosed with diabetes as part of this study, only 25 (11%) had previously been tested for diabetes.

Of the participants for whom we had full data, the proportion with two or more of hypertension, diabetes, and obesity were 68 (1%) of 4998 rural men, 246 (4%) of 6864 rural women, 155 (4%) of 3939 urban men, and 519 (7%) of 7516 urban women (table 2). Few participants had all three conditions (table 2). Prevalence increased with age more steeply in urban than in rural populations (figure 2). Hypertension and obesity was the most common combination of comorbidities for all groups except rural men, in whom diabetes and hypertension was the most common combination (albeit at a lower prevalence than was observed in urban-dwelling men). The magnitude of the association of urban residence, age, wealth, and education with multimorbidity was much larger than was the association of these factors with individual conditions (appendix p 12). In participants with diabetes, hypertension was greater than 50% but obesity was highly prevalent only in women (appendix p 13). Compared with those without a diabetes or hypertension diagnosis in the study, those who self-reported a previous diagnosis of diabetes or hypertension and treatment had an increased risk for being previously diagnosed for the other condition and an increased risk for being on treatment (appendix p 14).

## Discussion

In this large cross-sectional study, representative of the wider population of Malawi, we show a high cumulative prevalence of cardiovascular risk factors—overweight and obesity, hypertension, diabetes, and multimorbidity—in both urban and rural Malawi. For a country that has a young population with increasing life expectancy,^31^ the already high burden of these conditions (even in adults aged 50 years or less, and the non-obese) portends high rates of cardiovascular disease-related morbidity and premature mortality in the future. Tobacco and alcohol consumption and physical inactivity are currently less prevalent in Malawi than in most high-income countries,^32^, ^33^ but their prevalence in Malawi appears to be increasing.^14^ Levels of tobacco and alcohol consumption are comparable to those observed in neighbouring countries,^33^ where sociocultural and demographic characteristics and burdens of chronic infection^34^ are similar and where our findings might have greatest application for informing health policy and interventions.

Evidence suggests urbanisation and associated lifestyle and environment changes are important drivers of chronic NCDs^35^, ^36^ and, indeed, all three of the conditions we studied were more prevalent in urban than in rural residents. Nonetheless, this increased risk was not uniform; the higher prevalence of diabetes in urban than in rural residents diverged sharply with increasing age, whereas age-specific and sex-specific prevalence of hypertension in rural residents approached that of their urban counterparts, consistent with findings from the wider region, suggesting that any urban–rural gap has largely disappeared.^37^ Similarly, the effect of increasing wealth had a greater effect on risk for diabetes and overweight or obesity than for hypertension. Most hypertension and diabetes remained undetected, more notably in the rural setting, as has been observed elsewhere in sub-Saharan Africa,^37^ and patterns in treatment and control varied by area of residence, age, and sex.

The magnitude of likely cardiometabolic risk we found, which is consistent with a growing body of regional evidence,^12^, ^13^ is particularly concerning given that Malawi is one of the poorest countries in the world. That obesity affects more women than men is consistent with reports from neighbouring countries^38^, ^39^ but the very high age-specific prevalence of overweight and obesity (ie, BMI ≥25 kg/m^2^) in urban women, which is comparable to those in high-income countries,^40^, ^41^ and the stronger association of overweight and obesity with increasing wealth observed in men compared with women, was unexpected. Comparison of our data with earlier data from Malawi^14^ suggest an increasing obesity trend, at younger ages and more evident in women, consistent with pooled regional data.^12^ Exploration of the nutritional, social, and broader influences driving this trend are beyond the scope of our study.

We found an increased risk of hypertension and diabetes at relatively low or normal BMI and in all ages, even in a setting with high mean adult BMI. This is in contrast to data from high-income countries, in which only 20% of individuals with hypertension or diabetes have normal BMI.^42^ The reasons for this discrepancy are unclear, but might relate to factors specific to this setting; fetal exposure to maternal undernutrition^43^ or suboptimal early childhood nutrition are thought to increase susceptibility to obesity and NCDs in adulthood.^44^ Sodium intake in urban and rural Malawi is also uniformly high.

Our finding of higher prevalence of diabetes in the urban study population corresponded to greater overweight and obesity and lower physical activity levels (although still exceeding WHO daily activity recommendations) in the urban residents than in rural residents, yet overall our diabetes prevalence estimate is somewhat lower than a recent regional estimate.^13^ Indeed, age-standardised diabetes prevalence varies widely, with higher estimates in northern compared with southern Africa.^13^ The variation between studies might in part reflect different definitions and methodology (ie, HbA_1c_, FBG, or oral glucose tolerance tests) for measuring diabetes. Despite use of identical definitions, we observed lower diabetes prevalence than a 2009 Malawi estimate,^14^ although no population interventions have taken place. Our lower prevalence might be reflective of the larger sample size, and possibly the use of laboratory testing rather than point-of-care testing.

Our study was sufficiently large to explore age-specific and rural and urban burdens of disease, and used rigorous, standardised protocols and quality control procedures for data collection, and we accounted for the age structure of populations in prevalence estimates to enable external comparisons. However, the study does have limitations. Our data are cross-sectional and rely on self-reported measures for some health and lifestyle factors, so we cannot preclude the possibility of recall bias and reverse causation, particularly for physical activity and smoking, whereby behavioural changes caused by poor health can distort associations between these health-related behaviours and outcomes. People's occupation and assets can change over time, although our analyses in broad groupings should have minimised this effect. Use of FBG or a self-reported diagnosis of diabetes will have resulted in some misclassification, although corroboration with oral glucose tolerance tests results and validation of self-reports by HbA_1c_ measures suggest the effect on estimates of diabetes prevalence were minimal. Single-occasion blood pressure measurements overestimate true hypertension; as previously reported, 23·4% of those initially diagnosed with hypertension in our study did not have persistently elevated blood pressure at later clinical review.^45^

Because we visited homes several times, we only missed 12% of eligible rural individuals, but the lower participation of urban residents (62%)—particularly in men—might have resulted in some selection bias due to non-random non-participation. We have previously discussed the effect of differences in area-specific response rates and outcome differences by ease of recruitment.^16^ Non-participation might also be associated with reduced health-care-seeking behaviour or greater access to private health care.

Our findings highlight potential areas for prevention and management interventions, and support use of a syndemic approach to tackle these inter-related conditions—understanding contextual and environment factors that promote the co-occurrence of cardiovascular risk factors and their contribution to worsening health outcomes.^46^ For example, our finding of higher cardiometabolic risk in the less poor and better educated participants must be interpreted in context; 60% of Malawians are in the world's poorest billion, yet we saw an increased risk of overweight and obesity, hypertension, and diabetes even in the less poor compared with the most poor. Approaches to raising living standards and better nutrition in very poor nations must be matched by changes in the agricultural and food industries and national infrastructure that provide a non-obesogenic environment.

We observed considerable levels of multimorbidity in elderly Malawians, and found that having a diagnosis of and treatment for diabetes or hypertension was associated with access to care (ie, medication) for the other condition. Nonetheless, a high proportion of hypertension and diabetes remains undetected and management in those already diagnosed is poor across the region, particularly in rural residents^37^ and in men (who have less active health-seeking behaviour for other chronic health conditions such as HIV^27^ and lower uptake of referrals into care following screening for hypertension and diabetes than do women).^45^ Current guidelines in Malawi^30^ recommend screening for hypertension and diabetes in overweight individuals, smokers, those who drink excess alcohol, individuals with a family history of hypertension or diabetes, and (for blood pressure) those aged over 40 years. Our results suggest this approach would miss a high proportion of cases. Furthermore, community screening and referral—even to well supported clinics—might not yield the outcomes expected; we have previously shown that only 60% of individuals took up referrals, and of participants with hypertension offered medication, less than half continued to attend clinical follow-up within the study at 1 year.^27^ Although urban residents are at higher risk of overweight or obesity, hypertension, and diabetes, insufficient access to care in rural areas, as shown in our study, suggests that the unmet need is universal, and health system interventions must be designed so that they can be adapted to different contexts.

Our age-specific prevalence estimates show that the risk of developing overweight or obesity, hypertension, or diabetes is evident even in young adults and therefore interventions earlier in the life course will be essential; however, immediate interventions to reduce obesity in urban women of any age are imperative. Our data highlight the need for mechanistic studies to understand better NCD phenotypes in Africa, particularly in regard to the early age of onset of hypertension and diabetes in relatively lean and young people. The extent to which cardiometabolic risk factors (particularly multimorbidity) affect future risk of cardiovascular events and mortality in African populations is not well understood. Indeed, large cohorts with detailed baseline data, as presented here, with follow-up of health events and vital statistics will be essential to inform scientific and medical understanding and management of individual risk through risk score calculation.
