# Supplementary material for: Prevalence of obesity, hypertension, and diabetes, and cascade of care in sub-Saharan Africa: a cross-sectional, population-based study in rural and urban Malawi
Source: Lancet Diabetes Endocrinol. 2018 Mar;6(3):208–22. doi: 10.1016/S2213-8587(17)30432-1 (PMC5835666; doi:10.1016/S2213-8587(17)30432-1)
Supplement: Supplementary appendix [file mmc1.pdf]

THE LANCET
Diabetes & Endocrinology

Supplementary appendix

This appendix formed part of the original submission and has been peer reviewed. We post it as supplied by the authors.

Supplement to: Price A J, Crampin A C, Amberbir A, et al. Prevalence of obesity, hypertension, and diabetes, and cascade of care in sub-Saharan Africa: a cross-sectional, population-based study in rural and urban Malawi. *Lancet Diabetes Endocrinol* 2018; published online Jan 19. http://dx.doi.org/10.1016/S2213-8587(17)30432-1.

Supplemental materials

The prevalence of obesity, hypertension and diabetes, and cascade of care in sub-Saharan Africa: a large population-based study in rural and urban Malawi

Contents

Suppl. Figure 1. Conceptual model of risk factors for cardiometabolic outcomes *(page 2)*

Suppl. Figure 2. Adult population distributions and participation proportion of a) rural and b) urban populations *(page 3)*

Suppl. Figure 3. Distribution of Body Mass Index by site, sex and age group *(page 4)*

Suppl. Figure 4. Distribution of blood pressure by site, sex and age group *(page 5)*

Suppl. Figure 5. Distribution of blood glucose by site, sex and age group *(page 6)*

Suppl. Table 1: Association of urbanicity and gender with other hypertension, diabetes and anthropometry indices *(page 7)*

Suppl. Table 2: Stratified analyses for overweight/obesity *(page 8)*

Suppl. Table 3: Stratified analyses for hypertension *(page 9)*

Suppl. Table 4: Hypertension: Screening, cascade of care, access to diagnostic test, diagnosis, medication and control *(page 10)*

Suppl. Table 5: Diabetes: Screening, cascade of care, access to diagnostic test, diagnosis, medication and control *(page 11)*

Suppl. Table 6: Associations of risk factors with multi-morbidity – age-adjusted and distal factor adjusted risk ratios *(page 12)*

Suppl. Table 7: Prevalence of co-morbidities amongst diabetic and hypertensive patients *(page 13)*

Suppl. Table 8: Risk ratios (RR) for associations between a previous diagnosis and treatment for hypertension or diabetes and a diagnosis and medication for the other condition *(page 14)*

Supplementary Figure 1. Conceptual model of risk factors for cardiometabolic outcomes

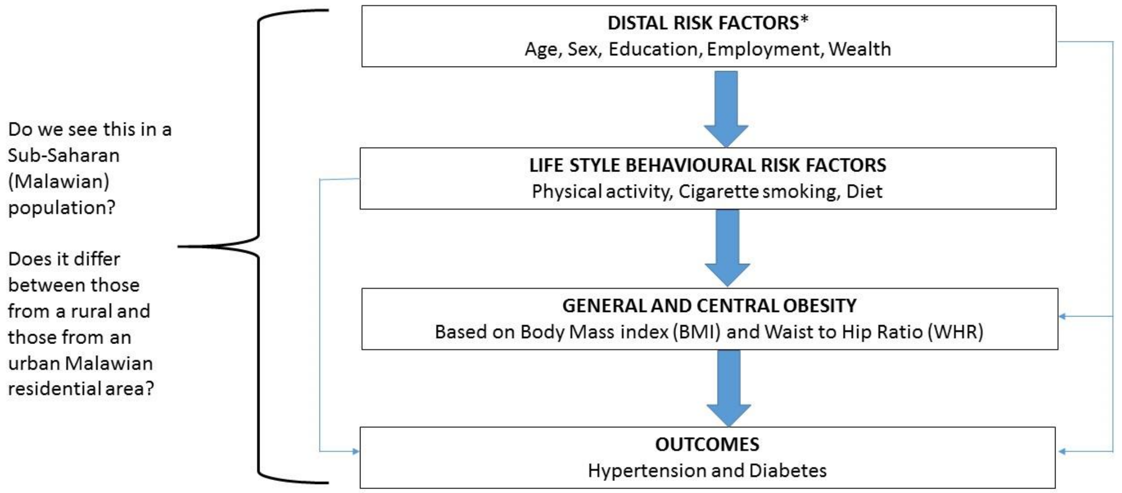

Supplementary Figure 2: Adult population distributions and participation proportion of a) rural and b) urban populations

Notes: Lowest age band consists of only two years. Ages of non-participants in urban populations are estimates.

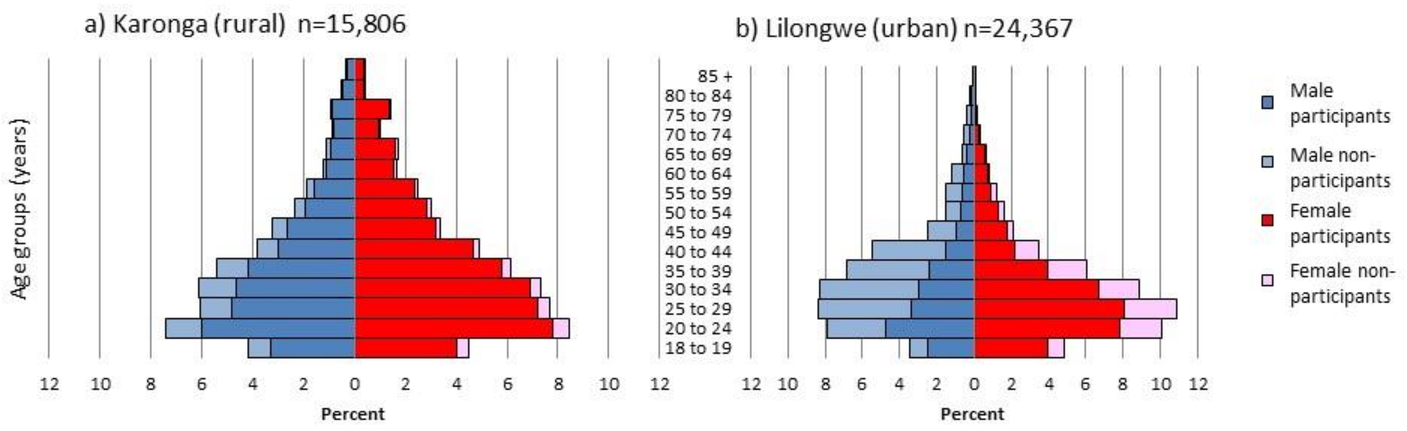

Supplementary Figure 3. Distribution of Body Mass Index by site, sex and age group

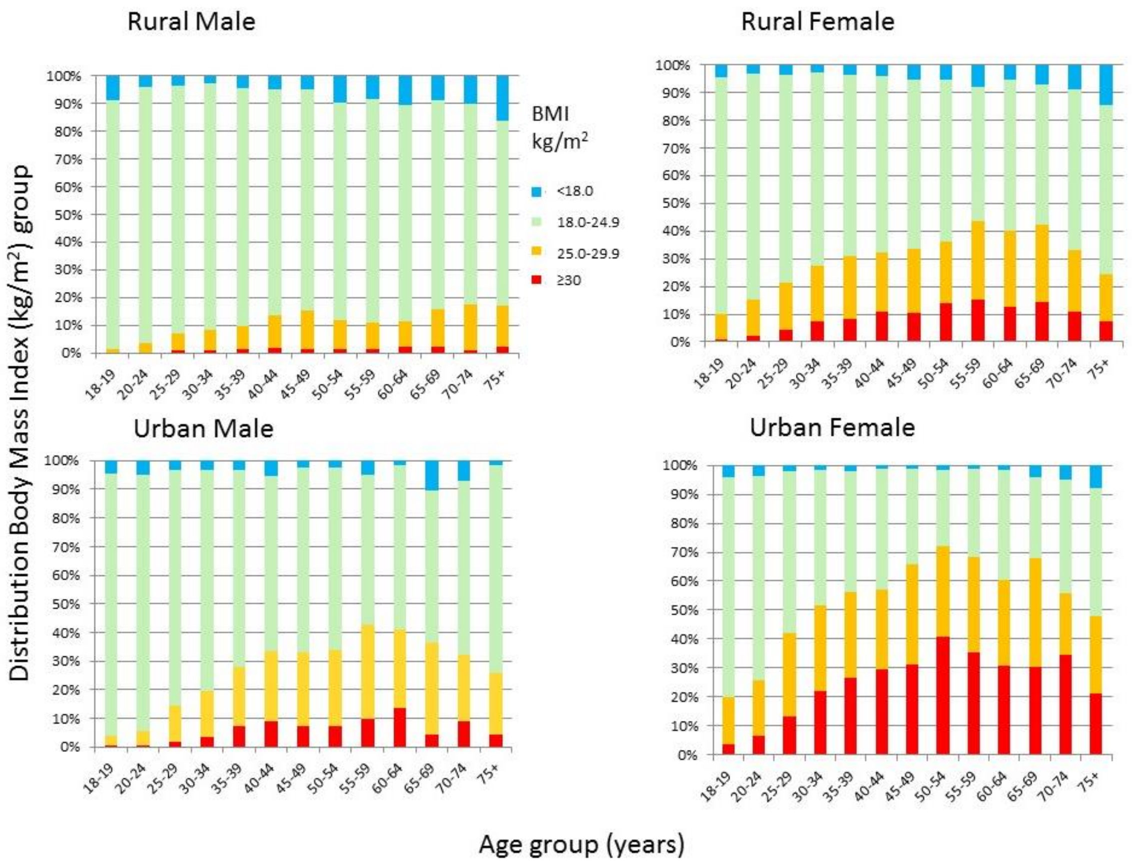

Supplementary Figure 4. Distribution of blood pressure by site, sex and age group

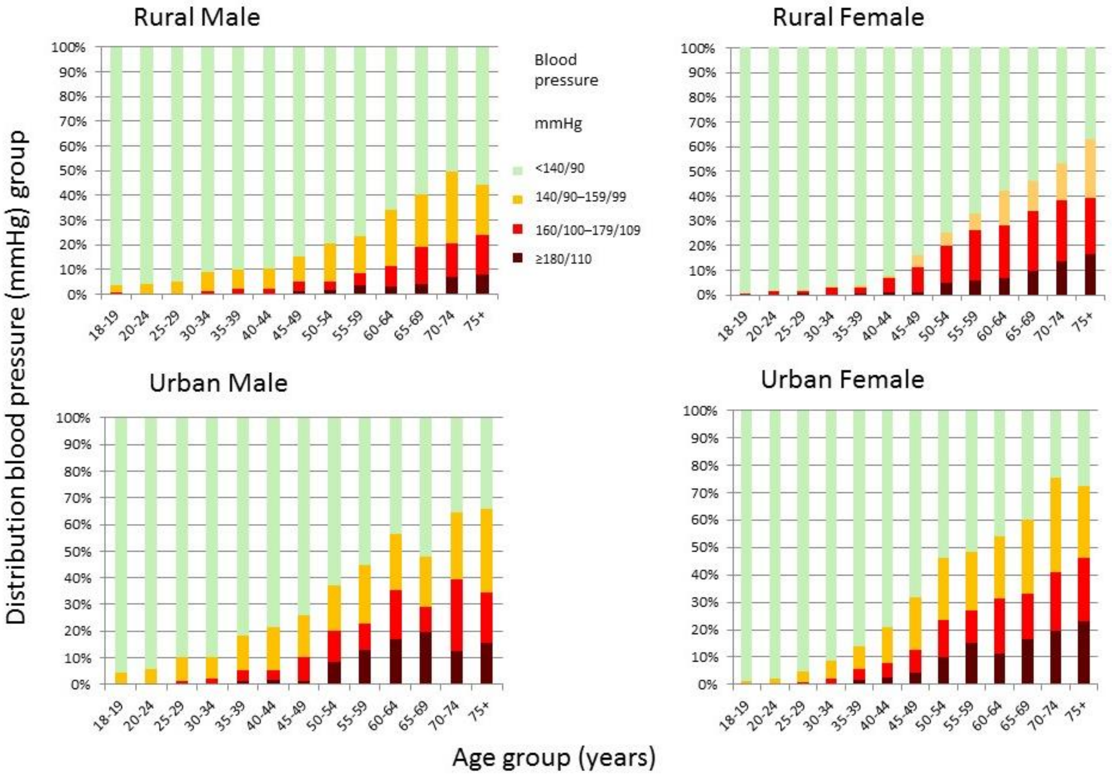

Supplementary Figure 5. Distribution of blood glucose by site, sex and age group

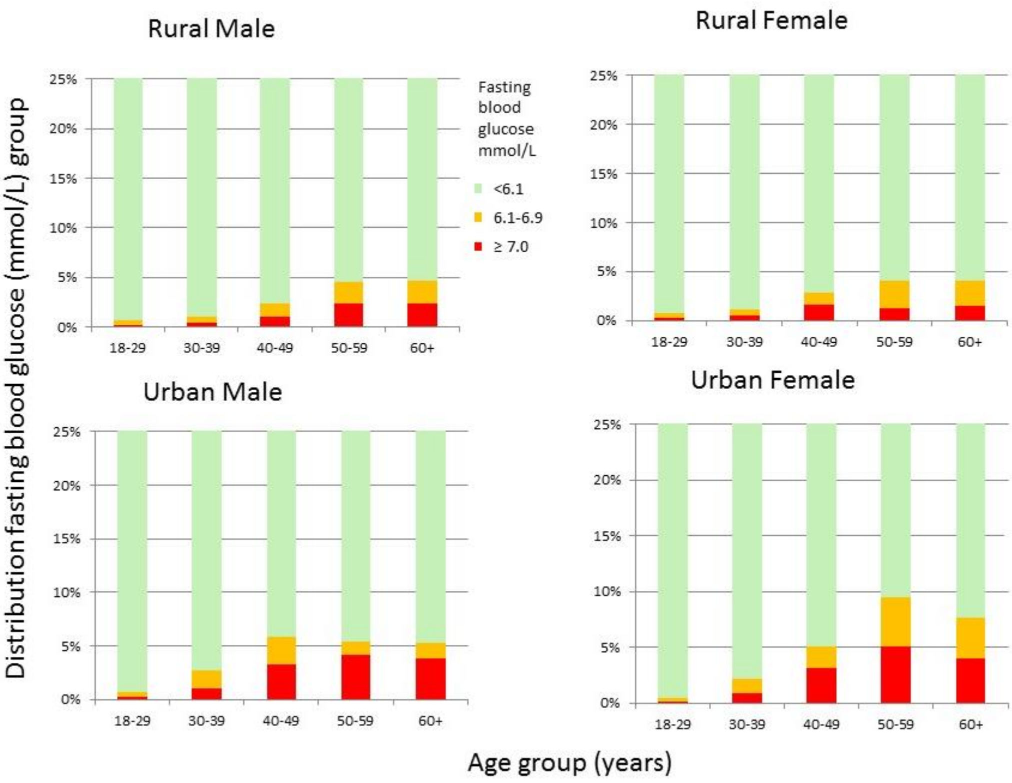

Supplementary Table 1. Association of urbanicity and gender with other hypertension, diabetes and anthropometry indices

	Rural[1]	Urban[1]	Male[2]	Female[2]
**Waist-to-hip ratio high**				
n (%)	5,158 (38·7%)	3,063 (21·1%)	2,030 (18·4%)	6,191 (36·9%)
Risk Ratio (Basic adjustment)	Ref	0·63 (0·60-0·65)	Ref	2·17 (2·08-2·26)
Adjusted Risk Ratio[3]	Ref	0·67 (0·64-0·71)	Ref	2·14 (2·05-2·23)
**Untreated Mod/Severe Hypertension[3]**				
n (%)	435 (3·3%)	513 (3·6%)	369 (3·4%)	579 (3·4%)
Risk Ratio (Basic adjustment)	Ref	2.10 (1·86-2·38)	Ref	1·14 (1·01-1·30)
Adjusted Risk Ratio	Ref	1·67 (1·42-1·99)	Ref	1·13 (0·99-1·30)
**On Hypertension medication**				
n (%)	509 (3·7%)	680 (4·5%)	307 (2·8%)	882 (5·0%)
Risk Ratio (Basic adjustment)	Ref	2·34 (2·11-2·60)	Ref	1·98 (1·75-2·23)
Adjusted Risk Ratio	Ref	1·37 (1·18-1·58)	Ref	2·51 (2·20-2·86)
**Undiagnosed diabetes[4]**				
n (%)	98 (0·8%)	135 (1·2%)	92 (1·0%)	141 (1·0%)
Risk Ratio (Basic adjustment)	Ref	2·06 (1·57-2·68)	Ref	0·95 (0·73-1·23)
Adjusted Risk Ratio	Ref	1·58 (1·05-2·37)	Ref	0·97 (0·93-1·28)
**On diabetes medication**				
n (%)	80 (0·58%)	147 (0·98%)	88 (0·80%)	139 (0·78%)
Risk Ratio (Basic adjustment)	Ref	2·90 (2·22-3·78)	Ref	1·03 (0·79-1·34)
Adjusted Risk Ratio	Ref	1·44 (0·97-2·13)	Ref	1·43 (1·08-1·90)

[1] Adjusted for age & sex
[2] Adjusted for age & site
[3] Untreated moderate or severe blood pressure systolic ≥ 160 mmHg and/or diastolic ≥100 mmHg and not on medication
[4] Undiagnosed diabetes: plasma glucose ≥7mmol/L and no prior diagnosis of diabetes

Supplementary Table 2. Stratified analyses for overweight/obesity

Overweight or obesity[1] Adjusted Risk Ratio[2] (95%CI)

Age – sex stratification

	Male < 30 years	Male 30-50 years	Male 50+ years	Female < 30 Years	Female 30-50 years	Female 50+ years
**Site**						
Karonga (rural)	Ref	Ref	Ref	Ref	Ref	Ref
Lilongwe (urban)	1·70 (1·22-2·38)	1·12 (0·94-1·33)	1·51 (1·20-1·89)	1·76 (1·52-2·03)	1·33 (0·21-1·46)	1·35 (1·21-1·51)
**Wealth quintiles**						
Poorest	Ref	Ref	Ref	Ref	Ref	Ref
2	1·65 (1·08-2·53)	1·38 (0·98-1·94)	1·02 (0·67-1·55)	1·13 (0·96-1·32)	1·16 (1·02-1·31)	1·11 (0·96-1·30)
3	1·37 (0·86-2·13)	2·39 (1·73-3·30)	1·40 (0·94-2·07)	1·25 (1·08-1·44)	1·31 (1·17-1·47)	1·40 (1·21-1·61)
4	1·53 (1·01-2·30)	2·90 (2·12-3·95)	1·57 (0·06-2·33)	1·34 (1·17-1·55)	1·51 (0·35-1·69)	1·40 (1·21-1·62)
Wealthiest	1·80 (1·17-2·77)	4·22 (3·08-5·80)	2·37 (1·60-3·51)	1·48 (1·28-1·72)	1·77 (1·58-1·97)	1·71 (1·47-1·97)
**Education**						
None	0.83 (0·14-0·55)	0·17 (0·03-1·00)	1·12 (0·68-1·86)	0·91 (0·66-1·26)	0·97 (0·83-1·14)	0.84 (0·74-0·96)
Standard1-5	0·67 (0·32-1·39)	0·75 (0·50-1·13)	1·03 (0·75-1·41)	1·01 (0·84-1·20)	0·97 (0·88-1·07)	0·88 (0·80-0·98)
Standard 6-8	Ref	Ref	Ref	Ref	Ref	Ref
Secondary	1·18 (0·86-1·63)	0·95 (0·79-1·14)	1·75 (1·39-2·20)	0·96 (0·87-1·06)	1·02 (0·95-1·09)	1·15 (1·03-1·27)
Post-secondary	2·09 (1·41-3·08)	1·36 (1·09-1·68)	1·71 (1·32-2·21)	0·98 (0·84-1·15)	1·13 (1·03-1·25)	1·16 (0·99-1·37)
**Employment**						
Not working	0·40 (0·29-0·55)	0·61 (0·44-0·84)	1·05 (0·83-1·32)	0·60 (0·53-0·68)	0·80 (0·71-0·90)	0.88 (0·74-0·97)
Housework	0·41 (0·21-0·82)	0·57 (0·33-0·98)	0·83 (0·52-1·33)	0·80 (0·72-0·90)	0·86 (0·80-0·92)	0·91 (0·81-1·02)
Subsistence	0·89 (0·58-1·36)	0·52 (0·41-0·67)	0·72 (0·52-0·98)	0·76 (0·63-0·91)	0·69 (0·62-0·77)	0·82 (0·72-0·95)
Self-employed	Ref	Ref	Ref	Ref	Ref	Ref
Salaried	0·96 (0·68-1·33)	1·00 (0·86-1·17)	0·93 (0·73-1·19)	0·80 (0·69-0·93)	0.87 0·80-0·95)	0·92 (0·76-1·10)

Site – sex stratification

	Rural Male	Rural Female	Urban Male	Urban Female
**Age group;** years				
18-29	Ref	Ref	Ref	Ref
30-39	2·06 (1·55-2·72)	1·72 (1·53-1·93)	2·24 (1·86-2·70)	1·58 (1·49-1·68)
40-49	3·00 (2·27-3·97)	1·97 (1·73-2·22)	3·07 (2·53-3·71)	1·75 (0·63-1·87)
50-59	2·54 (1·83-3·53)	2·48 (2·19-2·82)	3·82 (3·12-4·69)	2·09 (1·94-2·25)
60-69	3·25 (2·26-4·67)	2·77 (2·40-3·21)	5·13 (4·13-6·36)	2·05 (1·85-2·26)
70+	5·05 (3·70-6·89)	1·97 (1·62-2·39)	5·35 (3·93-7·28)	1·77 (1·50-2·09)
**Wealth quintiles**				
Poorest	Ref	Ref	Ref	Ref
2	1·24 (0·94-1·65)	1·11 (0·99-1·24)	1·31 (0·90-1·90)	1·16 (1·02-1·30)
3	1·78 (1·34-2·37)	1·25 (1·11-1·40)	1·71 (1·22-2·39)	1·32 (1·18-1·46)
4	1·66 (1·24-2·24)	1·37 (1·22-1·55)	2·22 (1·61-3·06)	1·46 (1·21-1·61)
Wealthiest	3·07 (2·26-4·17	1·62 (1·41-1·86)	2·71 (1·97-3·73)	1·67 (1·51-1·85)
**Education**				
None	0·72 (0·37-1·39)	0.89 (0·75-1·03)	0·74 (0·41-1·33)	0.91 (0·82-1·03)
Standard1-5	0·78 (0·57-1·06)	0.90 (0·80-0·99)	0·80 (0·56-1·16)	0.99 0·91-1·07)
Standard 6-8	Ref	Ref	Ref	Ref
Secondary	1·20 (0·99-1·45)	1·04 (0·94-1·15)	1·25 (1·03-1·51)	1·00 (0·94-1·06)
Post-secondary	0·96 (0·65-1·41)	0·91 (0·67-1·24)	1·96 (1·60-2·40)	1·09 (1·00-1·18)
**Employment**				
Not working	0·70 (0·52-0·95)	0·75 (0·63-0·88)	0·50 (0·41-0·60)	0·68 (0·63-0·74)
Housework	0·54 (0·19-1·49)	0·72 (0·62-0·84)	0·53 (0·38-0·73)	0·88 (0·83-0·93)
Subsistence	0·65 (0·52-0·82)	0·72 (0·65-0·80)	1·22 (0·67-2·22)	0·75 (0·55-1·04)
Self-employed	Ref	Ref	Ref	Ref
Salaried	1·27 (0·97-1·65)	1·08 (0·87-1·32)	0·90 (0·78-1·03)	0·85 (0·79-0·92)

[1]BMI ≥25kg/m^2

[2]Adjusted for distal factors; age, site (urban/rural), wealth, education, occupation (see Suppl. Figure 1. Conceptual model of risk factors for cardiometabolic outcomes (web appendix page 2)).

Supplementary Table 3. Stratified analyses for hypertension

Hypertension[1] Adjusted Risk Ratio[2](95%CI)						
**Age – sex stratification**						
	**Male < 30 years**	**Male 30-50 years**	**Male 50+ years**	**Female < 30 Years**	**Female 30-50 years**	**Female 50+ years**
**Site**						
Karonga (rural)	Ref	Ref	Ref	Ref	Ref	Ref
Lilongwe (urban)	1·78 (1·27-2·49)	1·31 (1·05-1·63)	1·22 (1·06-1·41)	1·32 (0·84-2·07)	1·56 (1·24-1·96)	1·10 (1·00-1·22)
**Wealth quintiles**						
Poorest	Ref	Ref	Ref	Ref	Ref	Ref
2	1·12 (0·76-1·66)	1·25 (0·95-1·66)	0·98 (0·79-1·21)	0·75 (0·45-1·26)	1·36 (1·03-1·80)	0·96 (0·86-1·09)
3	1·23 (0·84-1·81)	1·38 (1·04-1·84)	1·13 (0·92-1·40)	1·02 (0·63-1·65)	1·59 (1·22-2·08)	1·03 (0·91-1·16)
4	0·82 (0·54-1·22)	1·41 (1·07-1·87)	1·26 (1·03-1·55)	1·35 (0·87-2·09)	1·84 (1·42-2·39)	1·14 (1·01-1·28)
Wealthiest	1·09 (0·72-1·63)	1·54 (1·14-2·08)	1·42 (1·16-1·78)	1·27 (0·77-2·10)	2·23 (1·72-2·89)	1·14 (0·99-1·30)
**Education**						
None	1·51 (0·35-6·57)	1·08 (0·49-2·37)	0.92 (0·68-1·21)	1·45 (0·53-4·00)	1·16 (0·84-1·61)	1·18 (1·06-1·31)
Standard1-5	0·93 (0·52-1·67)	0·94 (0·65-1·37)	1·03 (0·87-1·22)	1·54 (0·89-2·67)	1·09 (0·88-1·34)	1·15 (1·04-1·26)
Standard 6-8	Ref	Ref	Ref	Ref	Ref	Ref
Secondary	0,92 (0·67-1·26)	0·99 (0·81-1·21)	1·16 (1·01-1·34)	1·05 (0·73-1·51)	0·97 (0·82-1·14)	1.01 (0·89-1·15)
Post-secondary	1·39 (0·91-2·14)	1·44 (1·11-1·88)	1·21 (1·01-1·44)	1·03 (0·59-1·80)	1·06 (0·82-1·37)	1·05 (0·84-1·30)
**Employment**						
Not working	0·58 (0·41-0·81)	0·82 (0·57-1·20)	1·71 (1·42-2·06)	0·66 (0·41-1·08)	0·88 (0·66-1·18)	1·51 (1·32-1·73)
Housework	0·83 (0·49-1·43)	0·62 (0·32-1·17)	1·36 (1·02-1·83)	0·95 (0·61-1·48)	1·02 (0·86-1·21)	1·16 (1·01-1·34)
Subsistence	0·83 (0·54-1·27)	0·96 (0·74-1·25)	1·15 (0·93-1·43)	0·59 (0·32-1·10)	0·73 (0·56-0·97)	0·93 (0·80-1·07)
Self-employed	Ref	Ref	Ref	Ref	Ref	Ref
Salaried	0·68 (0·46-1·01)	1·05 (0·86-1·28)	1·00 (0·81-1·24)	1·20 (0·69-2·09)	0·95 (0·77-1·17)	1·20 (0·96-1·50)
**Site – age stratification**						
	**Rural < 30 years**	**Rural 30-50 years**	**Rural 50+ years**	**Urban < 30 Years**	**Urban 30-50 years**	**Urban 50+ years**
**Sex**						
Male	Ref	Ref	Ref	Ref	Ref	Ref
Female	0·33 (0·23-0·47)	0·65 (0·54-0·78)	1·30 (1·18-1·44)	0·43 (0·34-0·54)	0·90 (0·79-1·04)	1·14 (1·03-1·26)
**Wealth quintiles**						
Poorest	Ref	Ref	Ref	Ref	Ref	Ref
2	1·16 (0·76-1·76)	1·23 (0·96-1·58)	0·95 (0·84-1·06)	0·72 (0·44-1·17)	1·44 (1·04-1·98)	1·09 (0·85-1·40)
3	1·10 (0·69-1·77)	1·35 (1·03-1·78)	1·05 (0·93-1·19)	1·16 (0·78-1·72)	1·72 (1·29-2·30)	1·27 (1·02-1·58)
4	0·87 (0·50-1·50)	1·40 (1·05-1·86)	1·02 (0·89-1·17)	1·10 (0·75-1·60)	1·97 (1·49-2·60)	1·61 (0·31-1·97)
Wealthiest	0·78 (0·36-1·70)	1·45 (1·00-2·08)	1·07 (0·89-1·28)	1·23 (0·84-1·82)	2·31 (1·75-3·05)	1·63 (1·33-2·01)
**Education**						
None	2·95 (0·81-10·75)	1·39 (0·81-2·37)	1·21 (1·07-1·38)	1·06 (0·38-2·95)	1·08 (0·75-1·55)	1·10 (0·95-1·27)
Standard1-5	1·30 (0·70-2·42)	0·96 (0·72-1·29)	1·15 (1·04-1·28)	1·10 (0·65-1·85)	1·11 (0·88-1·41)	1·03 (0·90-1·17)
Standard 6-8	Ref	Ref	Ref	Ref	Ref	Ref
Secondary	1·13 (0·79-1·60)	0·95 (0·77-1·17)	1·14 (098-1·32)	0·88 (0·64-1·20)	0·98 (0·83-1·16)	1.02 (0·91-1·15)
Post-secondary	2·38 (0·79-7·15)	1·37 (0·83-2·27)	1·67 (1·28-2·18)	1·09 (0·74-1·60)	1·18 (0·96-1·44)	1·03 (0·89-1·20)
**Employment**						
Not working	0·75 (0·44-1·28)	0·82 (0·48-1·42)	1·71 (1·42-2·06)	0·53 (0·39-0·73)	0·88 (0·68-1·13)	1·50 (1·31-·1·72)
Housework	1·84 (0·89-3·79)	0·94 (0·60-1·46)	1·24 (0·99-1·54)	0·71 (0·51-0·99)	1·05 (0·89-1·24)	1·21 (1·05-1·40)
Subsistence	0·93 (0·57-1·51)	0·78 (0·62-0·97)	1·05 (0·88-1·26)	1·00 (0·16-6·25)	1·47 (0·80-2·68)	1·00 (0·64-1·55)
Self-employed	Ref	Ref	Ref	Ref	Ref	Ref
Salaried	0·86 (0·32-2·30)	0.93 0·66-1·31)	1·03 (0·78-1·36)	0·75 (0·53-1·07)	1·01 (1·01 (0·87-1·19)	1·01 (0·85-1·20)

[1]Systolic ≥140mmHg and/or diastolic ≥90mmHg and/or on antihypertensive medication

[2]Adjusted for distal factors; age, site (urban/rural), wealth, education, occupation (see Suppl. Figure 1. Conceptual model of risk factors for cardiometabolic outcomes (web appendix page 2)).

Supplementary Table 4: Hypertension: Screening, cascade of care, access to diagnostic testing, a previous diagnosis, medication and control

	Total	Rural Male	Rural Female	Urban Male	Urban Female
**Of individuals aged ≥40 years N=8,552**					
Blood pressure measured ever	3,564/8,552<br>41·7%	378/2,180<br>17·3%	998/3,061<br>32·6%	742/1,288<br>57·6%	1,446/2,023<br>71·5%
Blood pressure measured ≥40 years	3,140/8,552<br>36·7%	354/2,180<br>16·2%	820/3,061<br>26·8%	682/1,288<br>53·0%	1,284/2,023<br>63·5%
**Of overweight and obese individuals N=8,702**					
Blood pressure measured ever	5,238/8,702<br>60·2%	132/536<br>24·6%	1,178/2,570<br>45·8%	611/8,702<br>63·5%	3,317/4,633<br>71·2%
**Of all current hypertensives, previously diagnosed[1] and on medication, and undiagnosed N=4,096**					
Blood pressure measured ever	2,277/4,096<br>55·6%	214/787<br>27·2%	483/1,101<br>43·9%	507/859<br>59·0%	1,073/1,349<br>79·5%
Previously diagnosed	1,708/4,096<br>41·7%	172/787<br>21·9%	482/1,101<br>43·8%	295/859<br>34·3%	759/1,349<br>56·3%
On medication	1,189/4,096<br>29·0%	127/787<br>16·1%	382/1,101<br>34·7%	180/859<br>21·0%	500/1,349<br>37·1%
**Of previously diagnosed hypertensives on regular medication and for whom a blood pressure measurement was available N=1,183[2]**					
Blood pressure controlled <140/90 mmHg	440/1,183<br>37·2%	41/127<br>32·3%	148/378<br>39·2%	50/179<br>27·9%	201/499<br>40·3%

[1]Excludes 849 people with a prior diagnosis of hypertension who were not on medication but did not have hypertension (BP>140/90) when measured in the study

[2] Excludes 6 people who did not have blood pressure measured during the study

Supplementary Table 5: Diabetes: Screening, cascade of care, access to diagnostic testing, a previous diagnosis, medication and control

	Total	Rural Male	Rural Female	Urban Male	Urban Female
**Of individuals aged ≥ 40 years and over N=8,552**					
Blood glucose test ever	858/8,552<br>10·0%	109/2,180<br>5·0%	140/3,061<br>4·6%	243/1,288<br>18·9%	366/2,023<br>18·1%
Blood glucose measured ≥40 years	784/8,552<br>9·2%	99/2,180<br>4·5%	130/3,061<br>4·3%	220/1,288<br>17·1%	335/2,023<br>16·6%
**Of overweight and obese individuals N=8,702**					
Blood glucose test ever	891/8,702<br>10·2%	42/536<br>7·8%	109/2,570<br>4·2%	191/963<br>19·8%	549/4,633<br>11·8%
**Of all individuals with diabetes, previously diagnosed & undiagnosed N=566**					
Blood glucose test ever[1]	304/566<br>53·7%	32/84<br>38·1%	53/124<br>42·7%	86/133<br>64·7%	133/225<br>59·1%
Previously diagnosed	333/566<br>58·8%	41/84<br>48·8%	69/124<br>55·7%	84/133<br>63·2%	139/225<br>61·8%
**Of all previously diagnosed diabetic individuals N=333**					
On regular medication	227/333<br>68·2%	33/41<br>80·5%	47/69<br>68·1%	55/84<br>65·5%	92/139<br>66·2%
Blood glucose controlled FBG <7·0 mmol/L	157/293<br>53·6%	21/37<br>56·8%	32/63<br>50·8%	44/69<br>63·8%	60/124<br>48·4%
**Of diagnosed diabetic individuals on regular medication N=227**					
Blood glucose controlled FBG <7·0 mmol/L	84/207<br>40·6%	14/29<br>48·3%	18/46<br>39·1%	25/47<br>53·2%	27/83<br>31·8%

[1]54 individuals previously diagnosed with diabetes reported never to have had a blood test for glucose, including 19 people on regular medication.

Supplementary Table 6: Associations of risk factors with multi-morbidity[1] – age-adjusted and distal factor adjusted risk ratios

	Males N=8,936		Females N=14,199	
**Characteristics**	**Age-adjusted Risk Ratio (95% CI)**	**Adjusted Risk Ratio[2] (95%CI)**	**Age-adjusted Risk Ratio (95% CI)**	**Adjusted Risk Ratio[2] (95%CI)**
**Site**				
Karonga (rural)	Ref	Ref	Ref	Ref
Lilongwe (urban)	4·00 (3·04-5·25)	1·91 (1·31-2·78)	3·11 (2·71-3·58)	1·43 (1·17-1·74)
**Age group;** years				
18-29	Ref	Re	Ref	Ref
30-39	8·66 (4·05-18·49)	9·81 (4·44-21·68)	5·89 (4·04-8·58)	5·72 (3·89-8·42)
40-49	16·10 (7·59-34·15)	17·71 (8·05-38·96)	16·34 (11·37-23·48)	17·44 (11·92-25·51)
50-59	28·41 (13·46-59·95	37·83 (17·45-82·03)	29·72 (20·78-42·51)	36·47 (25·11-52·98)
60-69	61·07 (29·37-127·00)	75·19 (36·16-156·35)	36·57 (25·36-52·73)	50·81 (34·94-73·88)
70+	31·57 (14·48-68·83)	72·70 (32·89-160·70)	31·76 (21·48-46·94)	51·76 (34·42-77·87)
**Wealth quintiles**				
Poorest	Ref	Ref	Ref	Ref
2	1·50 (0·70-3·24)	1·27 (0·59-2·76)	1·62 (1·16-2·29)	1·64 (1·17-2·31)
3	2·34 (1·11-4·91)	1·59 (0·75-3·37)	2·62 (1·91-3·61)	2·19 (1·59-3·03)
4	4·91 (2·44-9·84)	2·56 (1·25-5·24)	4·56 (3·39-6·12)	3·24 (2·40-4·38)
Wealthiest	10·54 (5·34-20·81)	3·91 (1·91-7·98)	6·48 (4·82-8·70)	3·96 (2·90-5·39)
**Education**				
None	<0·001 (0·001-<0·001)	<0·001 (0·001-<0·001)	0.82 (0·63-1·08)	0·95 (0·72-1·25)
Standard 1-5	0·60 (0·30-1·19)	0·65 (0·33-1·30)	0·85 (0·77-1·16)	1·07 (0·88-1·30)
Standard 5-8	Ref	Ref	Ref	Ref
Secondary	2·44 (1·70-3·51)	1.67 (1·13-2·46)	1·88 (2·59-3·99)	1·16 (0·96-1·39)
Post-secondary	7·06 (4·90-10·12)	2·90 (1·89-4·44)	3·21 (2·59-3·99)	1·46 (1·11-1·93)
**Employment**				
Not working	1·17 (0·80-1·71)	0·80 (0·54-1·18)	0·87 (0·68-1·11)	0·74 (0·58-0·96)
Housework	0·66 (0·29-1·53)	0·66 (0·29-1·48)	0·96 (0·79-1·16)	0·88 (0·72-1·07)
Subsistence	0·29 (0·19-0·44)	0·74 (0·45-1·21)	0·30 (0·24-0·38)	0·50 (0·38-0·65)
Self-employed	Ref	Ref	Ref	Ref
Salaried	1·08 (0·75-1·55)	0·73 (0·50-1·05)	1·36 (1·08-1·72)	0·87 (0·67-1·15)
**Physical activity**				
Did not meet	2·58 (1·92-3·47)	1·23 (1·01-1·51)	1·68 (1·29-2·20)	1·31 (1·00-1·73)
Met recommendation	Ref	Ref	Ref	Ref
**Smoking**				
Not current	Ref	Ref	Ref	Ref
Current	0·26 (0·13-0·50)	0·47 (0·24-0·92)	0·33 (0·05-2·21)	0·46 (0·08-2·75)
**Alcohol**				
Not in last year	Ref	Ref	Ref	Ref
In last year	0·73 (0·55-0·96)	0·98 (0·74-1·29)	1·84 (1·43-2·37)	1·84 (1·42-2·38)
**Sugary drinks intake**				
<6 tsps/day	Ref	Ref	Ref	Ref
≥6 tsps/day	0·86 (0·64-1·34)	0·74 (0·56-0·99)	1·09 (0·93-1·27)	0·93 (0·79-1·09)

[1] Multi-morbidity is defined as more than one concurrent condition of hypertension (Systolic ≥140mmHg and/or diastolic ≥90mmHg and/or on antihypertensive medication), diabetes (FBG ≥7·0mmol/L, or a previous self-reported diagnosis by a health professional, whether or not on medication) or obesity (BMI ≥30kg/m^2)

[2]Adjusted for distal factors; age, site, wealth, education, occupation (see Suppl. Figure 1. Conceptual model of risk factors for cardiometabolic outcomes (web appendix page 2)

Supplementary Table 7: Prevalence of co-morbidities amongst diabetic and hypertensive participants

	Rural Male n/N (%)	Rural Female n/N (%)	Urban Male n/N (%)	Urban Female n/N (%)
**Diabetic participants**				
**Hypertension**	44/84 (52·4)	64/124 (51·6)	79/133 (59·4)	138/225 (61·3)
**Obesity**	5/84 (6·0)	42/120 (35·0)	23/133 (17·3)	120/219 (54·8)
**Hypertensive participants**				
**Diabetes**	44/677 (6·5)	64/941 (6·8)	79/670 (11·8)	138/1,134 (12·2)
**Obesity**	33/781 (4·2)	210/1,073 (19·6)	106/858 (12·4)	504/1,329 (37·9)

Supplementary Table 8: Risk ratios (RR) for associations between a previous diagnosis and treatment for hypertension or diabetes and a diagnosis and medication for the other condition

**Hypertension status in study**	**RR of previous diabetes diagnosis**<br><br>**N =566**	**RR of already being on medication in those with previous diabetes diagnosis**<br>**N=333**
	**Adjusted RR[1] (95% CI)**	**Adjusted RR[1] (95% CI)**
No hypertension<br>Hypertension; previously undiagnosed<br>Previously diagnosed hypertension; no medication<br>Previously diagnosed hypertension; on medication	Ref<br>0·57 (0·39-0·82)<br>1·23 (0·99-1·54)<br>1·35 (1·12-1·64)	Ref<br>0·91 (0·65-1·26)<br>0·62 (0·42-0·92)<br>1·21 (1·00-1·47)
**Diabetes status in study**	**RR of hypertension diagnosis**<br><br>**N=3422**	**RR of already being on medication in those with a previous hypertension diagnosis**<br>**N=2216**
	**Adjusted RR[1] (95% CI)**	**Adjusted RR[1] (95% CI)**
No diabetes<br>Diabetes; previously undiagnosed<br>Previously diagnosed diabetes; no medication<br>Previously diagnosed diabetes; on medication	Ref<br>1·16 (0·98-1·36)<br>1·80 (1·53-2·11)<br>1·83 (1·69-1·99)	Ref<br>1·38 (1·17-1·63)<br>1·05 (0·79-1·41)<br>1·61 (1·48-1·75)

[1]Adjusted for site, sex and age
